# Design, Synthesis and Biological Evaluation of Novel SN38 Based Albumin-Binding Peptide–Drug Conjugates in Pancreatic Ductal Adenocarcinoma

**DOI:** 10.3390/pharmaceutics18081028

**Published:** 2026-08-19

**Authors:** Yi Su, Yingxin Lu, Jiahui Yu, Chen Jin, Yi Chen, Wei Lu, Jian Ding

**Affiliations:** 1 School of Chinese Materia Medica, Nanjing University of Chinese Medicine, Nanjing 210023, China; 2 State Key Laboratory of Drug Research, Shanghai Institute of Materia Medica, Chinese Academy of Sciences, Shanghai 201203, China; 3 School of Chemistry and Molecular Engineering, East China Normal University, 3663 North Zhongshan Road, Shanghai 200062, China; 4 Shanghai Tekanbio Pharm-Tech Co., Ltd., Room 4001, Floor 4, Unit 3, Building 8, No. 160, Basheng Road, China (Shanghai) Pilot Free Trade Zone, Shanghai 200131, China; jinc@tekanbio.com

**Keywords:** extra domain B fibronectin, albumin-binding, ZD2 peptide, pancreatic ductal adenocarcinoma

## Abstract

**Background and Objective:** Pancreatic ductal adenocarcinoma (PDAC) is characterized by dense extracellular matrix (ECM) with overexpression of extra domain B fibronectin (EDB-FN). **Methods:** Herein, we investigated novel albumin-binding peptide–drug conjugates, **Mc-ZD2-SN38** and **SSC-ZD2-SN38**, which integrate the prolonged circulation properties of albumin with the active targeting capability of the ZD2 peptide toward EDB-FN. Covalent and noncovalent albumin-binding strategies extended the half-life by approximately 100-fold and 15-fold, respectively, compared with the non-albumin-binding peptide–drug conjugate (PDC) (**ZD2-SN38**). **Results:** Notably, ZD2-mediated targeting markedly reduced payload accumulation in major organs, resulting in an improved safety profile. In a BxPC-3 xenograft model, **Mc-ZD2-SN38** and **SSC-ZD2-SN38** demonstrated potent and sustained antitumor efficacy. **Conclusions:** These findings establish a long circulation and active targeting drug delivery strategy, providing an ideal framework for the development of PDCs with enhanced therapeutic indices.

## 1. Introduction

The tumor microenvironment (TME) of pancreatic ductal adenocarcinoma (PDAC) represents a central factor contributing to its extreme malignancy, therapeutic resistance, and poor prognosis [1,2,3]. The TME of PDAC is characterized by a high stromal-to-tumor cell ratio, dense extracellular matrix (ECM) deposition with elevated interstitial fluid pressure, and poor vascularization [4,5]. PDAC tumor tissues aberrantly secrete ECM proteins such as fibronectin (FN), which drive tumor progression and metastasis, impede drug penetration, and promote chemotherapeutic resistance.

The extra domain B of fibronectin (EDB-FN) is overexpressed in various solid tumors, such as breast cancer, prostate cancer and pancreatic cancer, which is currently recognized as one of the most promising targets for tumor-specific drug delivery [6]. Several ligands have been developed, including ZD2 peptides, EDBp peptides and antibodies such as L19 [7,8]. The Lu group first developed ZD2 linear peptide (sequence: TVRTSAD) and cyclic peptide (sequence: CTVRTSADC) with high affinity for EDB-FN, which have been investigated as targeting moieties for both contrast agents and radio therapy in prostate, pancreatic, breast, and liver cancers [9,10,11,12]. ZD2-peptide-based contrast agents enable effective detection of early-stage, low-grade, indolent tumors, demonstrating potential for early cancer screening. The Tiwari group constructed a PDC by tandem fusion of cyclic ZD2 peptide with a cyclic cell penetrating peptide (cCPP), conjugated with the payload cabazitaxel (CBT) [13]. In this design, the cyclic ZD2 peptide first targets EDB-FN in the TME, while cCPP facilitates transmembrane penetration, thereby delivering the payload into tumor cells to exert cytotoxic effects. The Fan group further optimized the ZD2 sequence, identifying the EDBp peptide (sequence: AVRTSAD) with 336-fold higher binding affinity relative to cyclic ZD2 [14]. Their [^18^F]-EDBp agent exhibited high tumor uptake, with PET imaging enabling clear delineation, achieving efficacy in surgical navigation, radionuclide imaging, and targeted radiotherapy of thyroid cancer. In tumor-bearing mouse models, the targeted radiotherapeutic [^177^Lu]-EDBp effectively inhibited tumor growth and significantly prolonged survival.

PDCs comprise tumor-targeting peptides that recognize receptors overexpressed on tumor cells or within the TME. PDCs offer several distinct advantages, including low molecular weight, improved tumor penetration, and minimal immunogenicity [15,16,17,18,19,20,21]. In PDAC, the highly dense ECM poses a major obstacle to effective drug delivery. Due to its low molecular weight, the ZD2 peptides demonstrate considerable potential for penetrating this restrictive ECM barrier [22]. However, PDCs undergo rapid renal clearance, resulting in insufficient tumor exposure and limited therapeutic efficacy [23,24]. Consequently, a suitable macromolecular carrier is required to overcome this pharmacokinetic limitation and prolong systemic circulation.

Albumin-binding strategies have been employed to extend circulation time and improve tumor accumulation [25,26,27,28,29]. In our previous work, albumin-binding drugs **M-g-SN38** and **S-g-SN38** were designed to achieve covalent and noncovalent albumin-binding, respectively. Both albumin-binding drugs effectively enhanced the pharmacokinetic profile of the payload, increased tumor uptake, and significantly improved therapeutic outcomes [30]. Based on these findings, in this study, we developed albumin-binding PDCs by integrating ZD2-peptide-mediated targeting with an albumin-binding drug delivery strategy.

**Figure 1:**
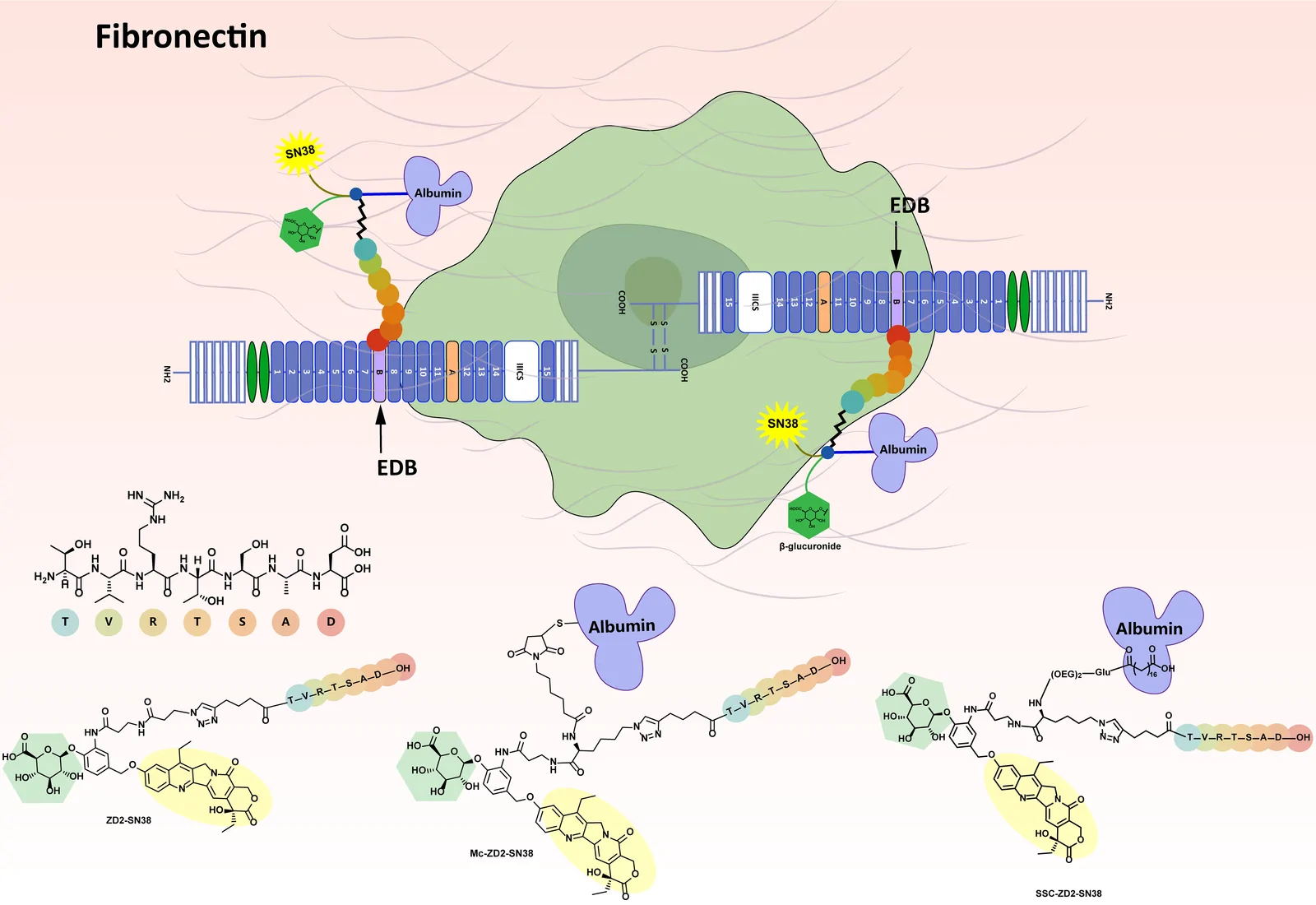
Schematic molecular structures of **Mc-ZD2-SN38**, **SSC-ZD2-SN38** and **ZD2-SN38** and their therapeutic strategies.

Both covalent and noncovalent albumin-binding PDCs were explored, named **Mc-ZD2-SN38** and **SSC-ZD2-SN38**, respectively. Albumin functions as a macromolecular carrier to mitigate rapid renal clearance, while the ZD2 peptide provides active targeting capability. The cytotoxic payload in these albumin-binding PDCs is SN38, which is connected via a self-immolative benzyl ether spacer to a β-glucuronide moiety. This configuration enables β-glucuronidase-triggered specific release of the **SN38** at the tumor site. **ZD2-SN38**, a PDC lacking an albumin-binding moiety, was synthesized as a control and compared with **Mc-ZD2-SN38** and **SSC-ZD2-SN38** in terms of in vitro and in vivo properties (Figure 1).

## 2. Experimental Section

### 2.1. Materials

All starting materials were commercially available and used without further purification. The reaction endpoints were determined by LC-MS. Normal-phase column chromatography was performed using a Biotage Isolera system (Biotage AB, Uppsala, Sweden) equipped with a Luknova SuperSep silica gel column (50 μm) (Luknova Inc., Mansfield, MA, USA). Reversed-phase chromatography was conducted on a Biotage Selekt system (Biotage AB, Sweden) with a SEPAFLASH spherical C18 column (20–45 μm, 100 Å) (Santai Technologies Inc., Changzhou, China). The purity of all compounds was determined to be >95% by HPLC analysis using an Agilent ZORBAX SB-C18 column (250 mm × 4.6 mm i.d., 5 μm) (Agilent Technologies Inc., Santa Clara, CA, USA). HPLC analyses were performed on a Shimadzu LC-20A system (Shimadzu Corporation, Kyoto, Japan) equipped with an SPD-M20A detector (Shimadzu Corporation, Japan). ^1^H NMR spectra were acquired on a Bruker DRX-400 spectrometer (400 MHz) (Bruker Corporation, Ettlingen, Germany) using CDCl_3_ or DMSO-*d*_6_ as solvents and TMS as an internal standard. Chemical shifts are reported as δ (ppm), and coupling constants as *J* (Hz). HRMS were obtained using a SCIEX X500 QTOF (SCIEX, Marlborough, MA, USA) or a Waters SYNAPT G2 ESI-TOF mass spectrometer (Waters Corporation, Milford, MA, USA).

### 2.2. HPLC Method

Agilent ZORBAX SB-C18 column was used for gradient analysis. Detection was performed at 254 nm and 360 nm, with the column temperature maintained at 40 °C and a flow rate of 1.0 mL/min. Mobile phase A consisted of an aqueous solution containing 0.1% TFA, and mobile phase B was ACN. The gradient program was as follows: 0–0.1 min, 5% B; 0.1–15 min, 5–95% B; 15.1–20 min, 95% B; 20.1–30 min, 5% B.

### 2.3. Chemistry Synthesis

#### 2.3.1. Synthesis of Compound **SC2**

Under a nitrogen atmosphere, Fmoc-l-lysine (3.5 g, 9.50 mmol), 1*H*-imidazole-1-sulfonyl azide hydrochloride (2.4 g, 11.40 mmol), K_2_CO_3_ (2.6 g, 19.00 mmol), and CuSO_4_·5H_2_O (24 mg, 0.10 mmol) were added to a flask and dissolved in a mixed solvent of 60 mL MeOH and 3 mL H_2_O. The mixture was stirred at room temperature for 12 h. The reaction was monitored by TLC until completion. Solids were removed and the organic solvent was evaporated. After pH adjusted to 4, the aqueous phase was extracted with ethyl acetate. The ethyl acetate phase was washed with saturated NaCl solution and dried over anhydrous NaSO_4_ for 5 h. After removal of the organic solvent, 3.70 g of compound **SC2** (pale-yellow oily liquid) was obtained in 100% yield. ^1^H NMR (400 MHz, CDCl_3_) δ 7.77 (d, *J* = 7.5 Hz, 2H), 7.60 (d, *J* = 7.2 Hz, 2H), 7.41 (t, *J* = 7.2 Hz, 2H), 7.32 (t, *J* = 7.4 Hz, 2H), 5.31 (d, *J* = 7.7 Hz, 1H), 4.81 (s, 1H), 4.56–4.40 (m, 3H), 4.23 (t, *J* = 6.7 Hz, 1H), 3.29 (t, *J* = 6.4 Hz, 2H), 1.93 (s, 1H), 1.80–1.70 (m, 1H), 1.63 (d, *J* = 6.5 Hz, 2H), 1.48 (s, 2H).

#### 2.3.2. Synthesis of Compound **SC3**

Under a nitrogen atmosphere, **SC2** (500 mg, 1.27 mmol) was dissolved in 5 mL of anhydrous ACN in a flask. *N*-hydroxysuccinimide (160 mg, 1.40 mmol) and EDCI (243 mg, 1.27 mmol) were added. The mixture was stirred at room temperature for 12 h. The reaction was monitored by TLC until completion. The solvent was removed to yield 394 mg of compound **SC3** (white solid) which was used directly in the subsequent reaction.

#### 2.3.3. Synthesis of Compound **3**

Under a nitrogen atmosphere, compound **1** (200 mg, 0.26 mmol) was dissolved in anhydrous DMF in a flask. DIPEA (68 μL, 0.53 mmol) and **SC3** (155 mg, 0.32 mmol) were added. The mixture was stirred at room temperature for 4 h. The reaction was monitored by LC-MS until completion. The solvent was removed to afford the crude product compound **2**. Compound **2** was dissolved in 4 mL of THF, followed by the addition of 1 mL diethylamine. The mixture was stirred at room temperature for 3 h. The reaction was monitored by LC-MS until completion. The solvent was removed and the crude product was purified by flash reversed-phase column chromatography (H_2_O with 0.1% TFA and ACN, ACN: 5%~50%). After removal of the organic solvent and lyophilization, 203 mg of compound **3** (yellow solid) was obtained in 84% yield. ^1^H NMR (400 MHz, DMSO-*d*_6_) δ 9.14 (s, 1H), 8.55 (s, 1H), 8.38 (s, 1H), 8.09 (d, *J* = 8.3 Hz, 4H), 7.59 (s, 1H), 7.55 (d, *J* = 9.2 Hz, 1H), 7.26 (d, *J* = 5.6 Hz, 2H), 7.15 (d, *J* = 8.3 Hz, 1H), 6.52 (s, 1H), 5.82 (s, 1H), 5.43 (s, 2H), 5.29 (d, *J* = 9.7 Hz, 4H), 4.89 (d, *J* = 7.3 Hz, 1H), 3.91 (d, *J* = 9.5 Hz, 1H), 3.70 (s, 2H), 3.36 (dd, *J* = 14.9, 8.2 Hz, 4H), 3.19 (t, *J* = 6.4 Hz, 4H), 2.64 (s, 2H), 1.94–1.79 (m, 2H), 1.66 (d, *J* = 7.6 Hz, 2H), 1.52 (d, *J* = 6.9 Hz, 1H), 1.46–1.38 (m, 2H), 1.25 (dd, *J* = 18.0, 10.5 Hz, 6H), 0.88 (t, *J* = 7.0 Hz, 3H).

#### 2.3.4. Synthesis of Compound **4**

Under a nitrogen atmosphere, ZD2 peptide (101 mg, 0.12 mmol) was dissolved in a mixed solvent of 1 mL MeOH and 1 mL H_2_O in a flask. CuSO_4_·5H_2_O (82 mg, 0.33 mmol) and sodium ascorbate (65 mg, 0.33 mmol) were added. After 0.5 h, compound **3** (100 mg, 0.11 mmol) was added, and the reaction was stirred at room temperature for 3 h. The reaction was monitored by LC-MS until completion. The solvent was removed and the crude product was purified by flash reversed-phase column chromatography (H_2_O with 0.1% TFA and ACN, ACN: 5%~35%). After removal of the organic solvent and lyophilization, 133 mg of compound **4** (yellow solid) was obtained in 69% yield. ^1^H NMR (400 MHz, DMSO-*d*_6_) δ 9.30 (s, 1H), 8.56 (s, 1H), 8.41 (s, 1H), 8.22 (d, *J* = 7.5 Hz, 1H), 8.16 (d, *J* = 7.5 Hz, 1H), 8.10 (s, 1H), 8.04–7.97 (m, 2H), 7.90 (d, *J* = 8.0 Hz, 1H), 7.73 (d, *J* = 11.8 Hz, 2H), 7.65–7.61 (m, 2H), 7.56 (d, *J* = 9.1 Hz, 2H), 7.28 (s, 3H), 7.19 (d, *J* = 8.0 Hz, 1H), 6.51 (s, 1H), 5.65 (s, 1H), 5.43 (s, 2H), 5.29 (d, *J* = 11.1 Hz, 4H), 5.20–4.99 (m, 3H), 4.60 (d, *J* = 6.8 Hz, 1H), 4.41 (d, *J* = 6.7 Hz, 2H), 4.30–4.25 (m, 4H), 4.20 (d, *J* = 8.7 Hz, 2H), 4.14 (dd, *J* = 16.2, 8.9 Hz, 5H), 3.96 (s, 1H), 3.71–3.66 (m, 2H), 3.58 (dd, *J* = 11.0, 4.6 Hz, 4H), 3.52 (s, 4H), 3.19 (d, *J* = 6.2 Hz, 4H), 3.08 (s, 3H), 2.67 (s, 2H), 2.57 (s, 4H), 2.34 (d, *J* = 13.6 Hz, 2H), 2.23 (s, 2H), 1.98–1.93 (m, 1H), 1.89–1.84 (m, 2H), 1.83–1.74 (m, 4H), 1.73–1.65 (m, 4H), 1.64–1.55 (m, 3H), 1.51 (s, 2H), 1.29–1.25 (m, 4H), 1.22 (d, *J* = 6.8 Hz, 4H), 1.02 (s, 6H), 0.83 (dd, *J* = 12.3, 5.3 Hz, 10H).

#### 2.3.5. Synthesis of Compound **Mc-ZD2-SN38**

Under a nitrogen atmosphere, compound **4** (100 mg, 0.06 mmol) was dissolved in H_2_O, followed by the addition of DIPEA (20 μL, 0.11 mmol) and EMCS (26 mg, 0.09 mmol) dissolved in ACN. The reaction mixture was stirred at room temperature for 1 h. The reaction was monitored by LC-MS until completion. The solvent was removed and the crude product was purified by flash reversed-phase column chromatography (H_2_O with 0.1% TFA and ACN, ACN: 5%~35%). After removal of the organic solvent and lyophilization, 88 mg of compound **Mc-ZD2-SN38** (yellow solid) was obtained in 79% yield. ^1^H NMR (400 MHz, DMSO-*d*_6_) δ 9.16 (s, 1H), 8.37 (s, 1H), 8.18 (t, *J* = 7.0 Hz, 2H), 8.09 (t, *J* = 8.0 Hz, 2H), 7.99 (s, 1H), 7.90 (d, *J* = 7.9 Hz, 1H), 7.83 (dd, *J* = 19.3, 10.6 Hz, 4H), 7.67 (d, *J* = 7.7 Hz, 1H), 7.62–7.52 (m, 3H), 7.36 (s, 1H), 7.29–7.23 (m, 2H), 7.14 (d, *J* = 8.5 Hz, 1H), 7.07 (d, *J* = 23.5 Hz, 1H), 6.99 (d, *J* = 9.7 Hz, 2H), 5.43 (s, 2H), 5.28 (d, *J* = 7.9 Hz, 4H), 4.89 (d, *J* = 6.7 Hz, 1H), 4.62–4.50 (m, 4H), 4.43–4.26 (m, 6H), 4.22 (d, *J* = 7.4 Hz, 4H), 4.06–3.96 (m, 3H), 3.92 (d, *J* = 9.6 Hz, 2H), 3.64 (d, *J* = 7.9 Hz, 6H), 3.43 (d, *J* = 14.0 Hz, 6H), 3.35 (s, 3H), 3.18 (d, *J* = 7.5 Hz, 2H), 3.10 (d, *J* = 4.8 Hz, 2H), 2.69 (dd, *J* = 16.5, 5.9 Hz, 1H), 2.58 (s, 3H), 2.23 (dd, *J* = 14.3, 6.8 Hz, 2H), 2.10–1.95 (m, 4H), 1.89–1.69 (m, 6H), 1.54 (d, *J* = 6.9 Hz, 6H), 1.48–1.39 (m, 6H), 1.27–1.21 (m, 6H), 1.19–1.10 (m, 4H), 1.03 (d, *J* = 5.9 Hz, 4H), 0.91–0.81 (m, 9H).

#### 2.3.6. Synthesis of Compound **SSC-ZD2-SN38**

Under a nitrogen atmosphere, compound **4** (100 mg, 0.06 mmol) was dissolved in anhydrous DMF in a flask. DIPEA (20 μL, 0.11 mmol) and Ste-rGlu-AEEA-AEEA-OSu (52 mg, 0.06 mmol) were added. The reaction mixture was stirred at room temperature for 1 h. The reaction was monitored by LC-MS until completion. The solvent was removed and the crude product was purified by flash reversed-phase column chromatography (H_2_O with 0.1% TFA and ACN, ACN: 5%~50%). After removal of the organic solvent and lyophilization, 127 mg of compound **SSC-ZD2-SN38** (yellow solid) was obtained in 90% yield. ^1^H NMR (400 MHz, DMSO-*d*_6_) δ 9.13 (s, 1H), 8.86 (s, 5H), 8.37 (s, 1H), 8.22–8.15 (m, 3H), 8.09 (d, *J* = 8.5 Hz, 2H), 8.04 (d, *J* = 7.6 Hz, 1H), 7.89 (t, *J* = 5.5 Hz, 1H), 7.87–7.78 (m, 4H), 7.77 (s, 1H), 7.71 (t, *J* = 5.5 Hz, 1H), 7.64 (d, *J* = 8.0 Hz, 1H), 7.60 (s, 2H), 7.55 (d, *J* = 9.3 Hz, 1H), 7.48 (s, 1H), 7.28 (s, 1H), 7.24 (d, *J* = 7.6 Hz, 1H), 7.14 (d, *J* = 8.6 Hz, 1H), 5.43 (s, 2H), 5.31 (s, 2H), 5.27 (s, 2H), 4.88 (d, *J* = 7.3 Hz, 1H), 4.58 (q, *J* = 6.7 Hz, 4H), 4.55–4.48 (m, 1H), 4.41–4.35 (m, 2H), 4.30 (d, *J* = 5.3 Hz, 5H), 4.22 (s, 3H), 4.13 (dd, *J* = 13.9, 7.3 Hz, 4H), 3.93 (s, 2H), 3.90 (s, 5H), 3.88 (s, 4H), 3.82 (s, 5H), 3.66–3.62 (m, 6H), 3.55 (s, 2H), 3.53 (s, 2H), 3.44 (s, 3H), 3.39 (s, 3H), 3.34 (s, 1H), 3.32–3.26 (m, 3H), 3.19 (s, 5H), 3.09 (d, *J* = 3.8 Hz, 2H), 2.68 (dd, *J* = 16.4, 5.9 Hz, 2H), 2.58 (s, 6H), 2.25 (d, *J* = 8.0 Hz, 1H), 2.18–2.08 (m, 6H), 1.98–1.84 (m, 4H), 1.81–1.66 (m, 7H), 1.52 (d, *J* = 6.9 Hz, 12H), 1.47 (s, 5H), 1.26 (s, 2H), 1.22 (s, 30H), 1.02 (d, *J* = 5.6 Hz, 6H), 0.89–0.80 (m, 10H).

#### 2.3.7. Synthesis of Compound **SC5**

Under a nitrogen atmosphere, 3-azidopropionic acid (500 mg, 4.34 mmol) was dissolved in 5 mL of anhydrous ACN in a flask. *N*-Hydroxysuccinimide (1.16 g, 6.51 mmol) and EDCI (1.25 g, 6.51 mmol) were added. The mixture was stirred at room temperature for 12 h. The reaction was monitored by TLC until completion. Purification by flash column chromatography on silica gel (hexane and ethyl acetate, ethyl acetate: 40%) followed by removal of the solvent afforded 698 mg of compound **SC5** (white solid), which was used directly in the subsequent reaction.

#### 2.3.8. Synthesis of Compound **5**

Under a nitrogen atmosphere, compound **1** (100 mg, 0.13 mmol) was dissolved in anhydrous DMF in a flask. DIPEA (46 μL, 0.26 mmol) and compound **SC5** (33 mg, 0.16 mmol) were added. The mixture was stirred at room temperature for 4 h. The reaction was monitored by LC-MS until completion. The solvent was removed and the crude product was purified by flash reversed-phase column chromatography (H_2_O with 0.1% TFA and ACN, ACN: 5%~50%) followed by lyophilization to yield 98 mg of compound **5** (yellow solid) in 87% yield. ^1^H NMR (400 MHz, DMSO-*d*_6_) δ 9.14 (s, 1H), 8.37 (s, 1H), 8.13 (t, *J* = 5.5 Hz, 1H), 8.07 (d, *J* = 9.1 Hz, 1H), 7.61–7.51 (m, 2H), 7.26 (d, *J* = 10.8 Hz, 2H), 7.14 (d, *J* = 8.4 Hz, 1H), 5.43 (s, 2H), 5.27 (d, *J* = 4.2 Hz, 4H), 4.89 (d, *J* = 7.4 Hz, 1H), 3.91 (d, *J* = 9.6 Hz, 1H), 3.43–3.32 (m, 11H), 3.16 (d, *J* = 6.9 Hz, 2H), 2.59 (t, *J* = 6.6 Hz, 2H), 2.38 (t, *J* = 6.4 Hz, 2H), 1.86 (dq, *J* = 14.0, 7.0 Hz, 2H), 1.53 (d, *J* = 7.0 Hz, 1H), 1.25 (t, *J* = 7.5 Hz, 3H), 0.88 (t, *J* = 7.3 Hz, 3H).

#### 2.3.9. Synthesis of Compound **ZD2-SN38**

Under a nitrogen atmosphere, ZD2 peptide (74 mg, 0.09 mmol) was dissolved in a mixed solvent of 1 mL MeOH and 1 mL H_2_O in a flask. CuSO_4_·5H_2_O (44 mg, 0.17 mmol) and sodium ascorbate (35 mg, 0.17 mmol) were added. After 0.5 h, compound **5** (50 mg, 0.06 mmol) was added, and the reaction was stirred at room temperature for 3 h. The reaction was monitored by LC-MS until completion. The solvent was removed and the crude product was purified by flash reversed-phase column chromatography (H_2_O with 0.1% TFA and ACN, ACN: 5%~35%). After removal of the organic solvent and lyophilization, 76 mg of **ZD2-SN38** (yellow solid) was obtained in 77% yield. ^1^H NMR (400 MHz, DMSO-*d*_6_) δ 9.13 (s, 1H), 8.37 (s, 1H), 8.28–8.07 (m, 5H), 7.85–7.78 (m, 3H), 7.58 (dd, *J* = 22.2, 8.8 Hz, 2H), 7.46 (s, 1H), 7.26 (d, *J* = 13.4 Hz, 2H), 7.14 (d, *J* = 8.1 Hz, 1H), 5.43 (s, 2H), 5.30 (d, *J* = 7.5 Hz, 4H), 4.89 (d, *J* = 7.0 Hz, 1H), 4.53 (dt, *J* = 22.1, 6.3 Hz, 3H), 4.40–4.18 (m, 5H), 4.05–3.93 (m, 2H), 3.91 (d, *J* = 9.2 Hz, 1H), 3.79 (s, 2H), 3.65–3.61 (m, 4H), 3.22–3.14 (m, 7H), 3.12–3.06 (m, 4H), 2.72–2.64 (m, 4H), 2.56 (d, *J* = 8.5 Hz, 6H), 2.36–2.14 (m, 6H), 2.02–1.65 (m, 9H), 1.52 (d, *J* = 6.8 Hz, 6H), 1.30–1.20 (m, 6H), 1.02 (d, *J* = 5.4 Hz, 6H), 0.91–0.78 (m, 10H).

### 2.4. Stability Evaluation

**Mc-ZD2-SN38**, **SSC-ZD2-SN38**, and **ZD2-SN38** were prepared at 1 mM in DMSO. A total of 2 μL of **Mc-ZD2-SN38**, **SSC-ZD2-SN38** or **ZD2-SN38** was added to 98 μL of human or murine plasma. The mixture samples were incubated in a shaking air bath at 37 °C. The above solutions were collected at day 0.25, 1, 4 and 7 followed by addition of 100 μL of cold ACN. Following vortex mixing and centrifugation, the supernatant was analyzed by HPLC for **SN38** quantification (HPLC method).

### 2.5. In Vitro β-Glucuronidase-Dependent Drug Release

**Mc-ZD2-SN38**, **SSC-ZD2-SN38**, and **ZD2-SN38** were prepared at 1 mM in DMSO. β-Glucuronidase (Sigma-Aldrich, St. Louis, MO, USA) was dissolved in phosphate buffer (pH 7.0) to prepare at a stock solution at 25 KU/mL. For the drug release assay, 10 μL of the enzyme stock solution (250 U) was diluted to 900 μL with phosphate buffer, followed by 100 μL of the **Mc-ZD2-SN38**, **SSC-ZD2-SN38** or **ZD2-SN38**. The mixtures were incubated in a shaking air bath at 37 °C. 40 μL of aliquots were withdrawn at predetermined time (0.2 min, 0.5 min, 1.0 min, 2.0 min, 3.0 min and 5.0 min) and quenched by addition of 160 μL of cold ACN. After vortex mixing, the samples were centrifuged, and the supernatant was subjected to HPLC analysis for **SN38** quantification (HPLC Method).

### 2.6. Cells, Animals, Proteins and Reagents

The BxPC-3 human pancreatic cancer cell line was obtained from the American Type Culture Collection (ATCC). Cells were cultured in RPMI-1640 medium supplemented with 10% fetal bovine serum (FBS) and 1% penicillin–streptomycin, and incubated in a humidified atmosphere containing 5% CO_2_ at 37 °C. Female BALB/c mice (6~8 weeks old) were purchased from Jihui Laboratory Animal Care Co., Ltd. (Shanghai, China) and Shanghai Institute of Materia Medica, Chinese Academy of Sciences. The animal study protocol was approved by the Institutional Review Board (or Ethics Committee) of Institutional Animal Care and Use Committee at East China Normal University (protocol no. m20251008, 30 October 2025) and Institutional Animal Care and Use Committee at Shanghai Institute of Materia Medica, Chinese Academy of Sciences (protocol no. 2024-06-DJ-96, 13 June 2024).

### 2.7. In Vitro CCK8 Assay

BxPC-3 cells were seeded at a density of 2 × 10^3^ cells per well in 96-well plates and incubated for 24 h. Two control groups were treated with irinotecan and **SN38**, respectively (3333.3 nM, 1111.1 nM, 370.4 nM, 123.5 nM, 41.2 nM, 13.7 nM, 4.6 nM and 1.5 nM). Test compounds (**Mc-ZD2-SN38**, **SSC-ZD2-SN38**, or **ZD2-SN38**) were added to the cells at equimolar concentrations in the presence or absence of β-glucuronidase and further incubated for 72 h. Cell viability was assessed using the CCK8 assay. IC50 values were calculated using GraphPad Prism 8 software.

### 2.8. Pharmacokinetic Study

Female BALB/c mice received a single intravenous dose of 25 mg/kg **irinotecan**, **Mc-ZD2-SN38**, **SSC-ZD2-SN38**, or **ZD2-SN38** via tail vein injection. At predetermined time points, 3 mice from each group were sacrificed and 200 μL plasma samples were collected.

Plasma samples from the **Mc-ZD2-SN38** group were divided into two aliquots. To determine free **SN38** concentrations, 100 μL of plasma was directly deproteinized by addition of 100 μL ACN, followed by vortex mixing and centrifugation to obtain the supernatant for analysis. To quantify total **SN38** (free plus albumin-binding conjugate), another 100 μL aliquot was incubated with 20 μL β-glucuronidase stock solution (25 KU/mL) at 37 °C for 24 h to hydrolyze the albumin conjugate. Subsequently, 80 μL ACN was added, and the mixture was subjected to vortex mixing and centrifugation to obtain the supernatant for analysis. Both supernatant aliquots were analyzed by HPLC and **SN38** concentrations were quantified based on standard calibration curves. The concentration of **Mc-ZD2-SN38** in plasma was calculated by subtracting the free **SN38** concentrations from the total **SN38** concentrations.

Plasma samples from the **SSC-ZD2-SN38**, **ZD2-SN38**, and irinotecan groups were deproteinized by addition of 200 μL ACN, followed by vortex mixing and centrifugation to obtain the supernatants for analysis. Concentrations of **SSC-ZD2-SN38**, **ZD2-SN38**, and irinotecan were quantified using respective standard calibration curves.

### 2.9. Biodistribution Study

Biodistribution studies were performed in female BALB/cA nude mice (4 weeks old) bearing subcutaneous BxPC-3 xenograft tumors. When tumor volumes reached approximately 250 mm^3^, mice were randomly assigned to 5 groups (*n* = 6). Mice received a single intravenous dose of 25 mg/kg **irinotecan**, **Mc-ZD2-SN38**, **SSC-ZD2-SN38**, **M-g-SN38**, or **ZD2-SN38** via tail vein injection. At 6 or 30 h post-injection, 3 mice from each group were euthanized, and the tumors and major organs were excised and washed with PBS (pH 7.4), weighed, and homogenized in 1000 μL of ACN. Following centrifugation, the supernatant was analyzed by HPLC (HPLC method), and **SN38** concentrations were quantified based on standard calibration curves. The percentage of injected dose (%ID) and percentage of injected dose per gram of tissue (%ID/g) were calculated using the following equations:


(1) %ID = (dose in tissue sample/injected dose)×100

(2) %ID/g = %ID/weight of tissue

### 2.10. In Vivo Animals Studies in BxPC-3 Xenograft Model

Female BALB/c nu/nu mice (3~4 weeks old) were housed in the Experimental Animal Center of the Shanghai Institute of Materia Medica under SPF conditions. BxPC-3 cells (5 × 10^6^) were inoculated subcutaneously into the right axilla of nude mice. Tumor tissues at the vigorous growth stage were dissected into fragments of approximately 1.5 mm^3^ and transplanted subcutaneously into the right axilla of recipient nude mice under sterile conditions. Tumor dimensions were measured using vernier calipers, and mice were randomly assigned to experimental groups once tumor volumes reached approximately 80 mm^3^.

Mice bearing BxPC-3 xenografts were randomized into 8 groups (vehicle *n* = 12, treatment group *n* = 6). Intravenous injections were administered according to the following dosing regimens: vehicle (solvent control), irinotecan (15 mg/kg), **Mc-ZD2-SN38** (26 mg/kg and 52 mg/kg), **SSC-ZD2-SN38** (33 mg/kg and 66 mg/kg), and **ZD2-SN38** (23 mg/kg and 46 mg/kg). Mice in the **Mc-ZD2-SN38**, **SSC-ZD2-SN38**, and **ZD2-SN38** groups received weekly intravenous injections via the tail vein for three consecutive weeks (three doses total). The irinotecan group was administered twice weekly for three weeks (six doses total). Following the cessation of dosing, mice were monitored for an additional 2 weeks until the end of the experiment (day 35). Tumor dimensions and body weights were recorded twice weekly throughout the experiment. All mice were euthanized after the experiment. Tumors and major organs were excised, rinsed with PBS, and collected for histological analysis by H&E staining.

V_0_ is the tumor volume at the time of randomization. V_t_ is the tumor volume at each measurement. T_RTV_ is RTV in the treatment group. C_RTV_ is the negative control group. The tumor volume (TV), relative tumor volume (RTV), and relative tumor proliferation rate (T/C) were calculated using the following equations:


(3) $$TV\;=\;length\times width^{2}/2$$

(4) $$RTV\;=\;V_{t}/V_{0}$$

(5) $$T/C(\%)\;=\;T_{RTV}/C_{RTV}\times 100\%$$

### 2.11. Histological Analysis

The tumors and major organs were fixed in 4% formaldehyde, embedded in paraffin, and sectioned to obtain 3 μm slices for H&E staining analysis. The H&E-stained tissues were imaged using an optical microscope.

### 2.12. Statistical Analysis

All experiments were performed in triplicate and data are presented as mean ± SD. Statistical significance was calculated using Two-way ANOVA followed by Dunnett’s post hoc test and Student’s *t*-test. Significant differences are indicated in the relevant figures as * *p* < 0.05, ** *p* < 0.01, or *** *p* < 0.001.

## 3. Results and Discussion

### 3.1. Design and Synthesis of PDCs

**Scheme 1:**
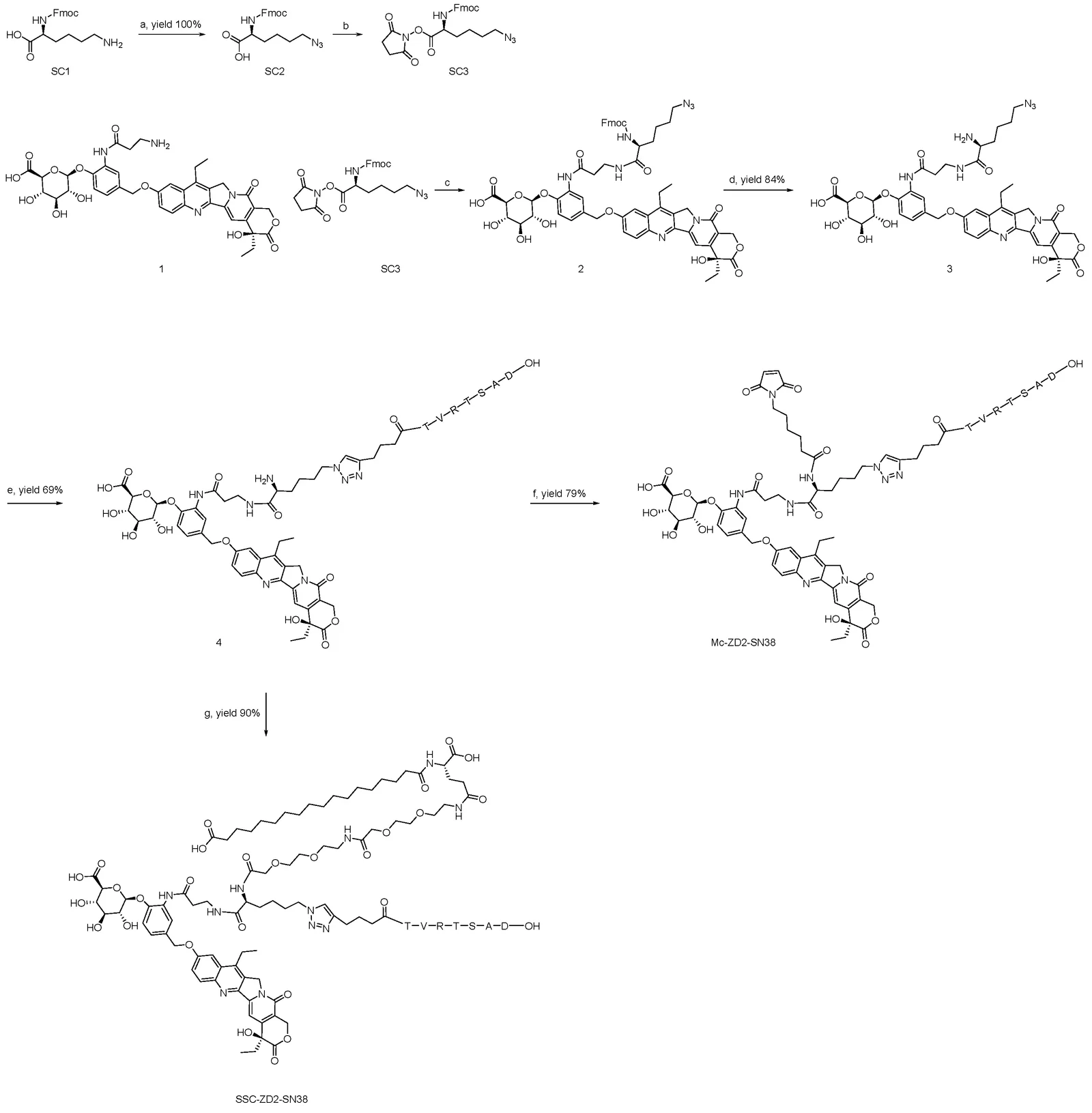
Reagents and Conditions: (a) 1H-Imidazole-1-sulfonyl azide hydrochloride, K_2_CO_3_, CuSO_4_·5H_2_O, MeOH, H_2_O, rt.; (b) *N*-Hydroxysuccinimide, EDCI, DCM, rt.; (c) DIPEA, DMF, rt.; (d) Diethylamine, THF, rt.; (e) ZD2 peptide, CuSO_4_·5H_2_O, Sodium ascorbate, MeOH, H_2_O, rt.; (f) EMCS, DIPEA, DMF, rt.; (g) HO-Ste-Glu-(OEG2-OSu)-OH, DIPEA, DMF, rt.

**Scheme 2:**
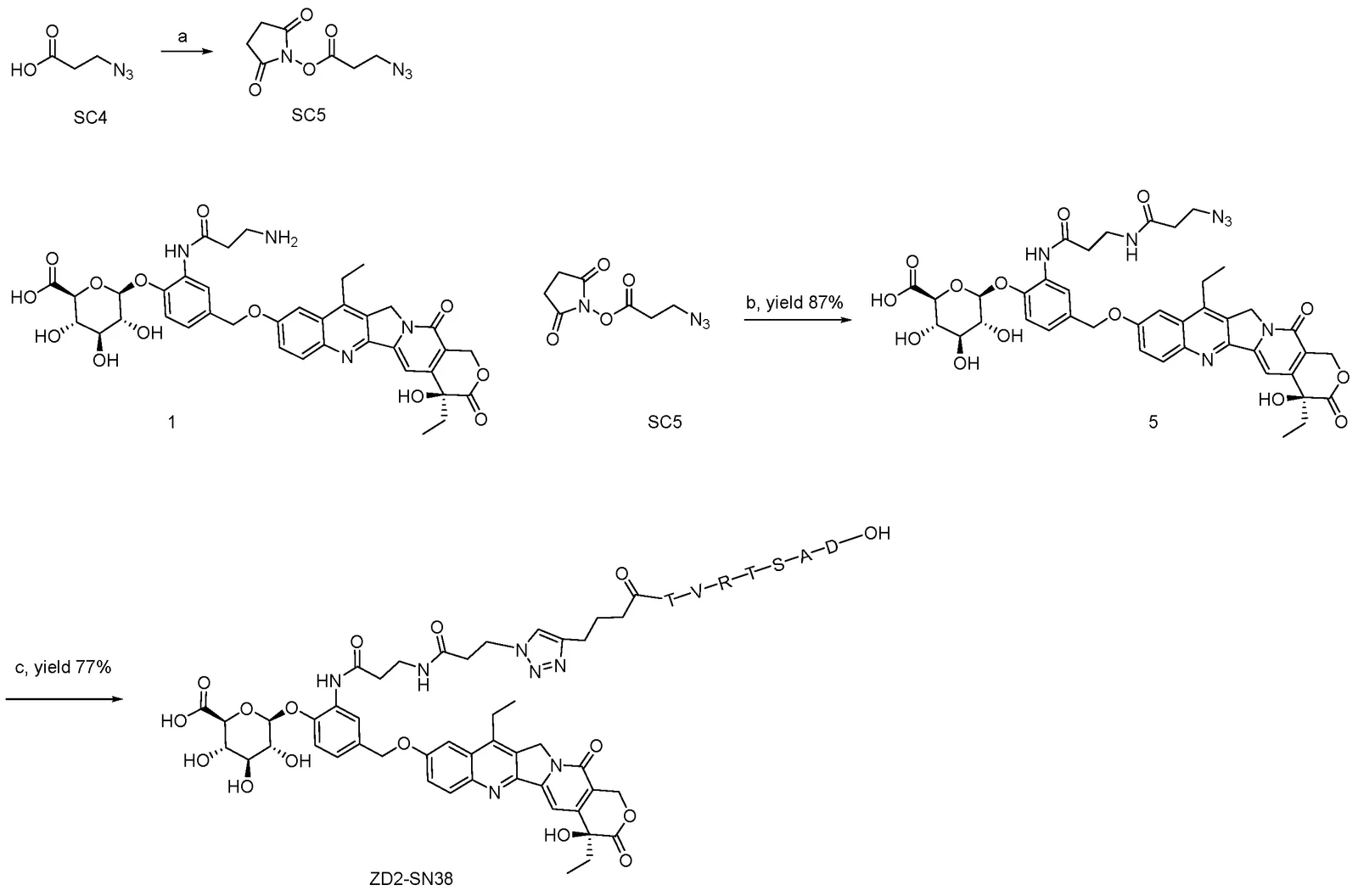
Reagents and Conditions: (a) *N*-Hydroxysuccinimide, EDCI, DCM, rt.; (b) DIPEA, DMF, rt.; (c) ZD2 peptide, CuSO_4_·5H_2_O, Sodium ascorbate, MeOH, H_2_O, rt.

Compound **1** was synthesized according to the reported procedure [30]. Using compound **1** as the starting material, three PDCs were prepared: **Mc-ZD2-SN38** and **SSC-ZD2-SN38**, which feature covalent and noncovalent albumin-binding moieties, respectively, and **ZD2-SN38**, which lacks an albumin-binding moiety. The target recognition capability of the ZD2 peptide (H_2_N-TVRTSAD-OH) is retained regardless of N-terminal extensions. The alkyne-functionalized ZD2 peptide, comprising the TVRTSAD sequence with a 5-hexynoic acid spacer for click chemistry [31], was obtained commercially. A trifunctional crosslinker (**SC3**) was designed to simultaneously link three components: the payload via amide bond formation, the alkyne-functionalized ZD2 peptide via click chemistry, and an albumin-binding moiety such as a maleimide or a semaglutide side chain (Scheme 1). A short spacer, 3-azidopropanoic acid, was designed to link the compound **1** with the alkyne-functionalized ZD2 peptide for the preparation of **ZD2-SN38** (Scheme 2).

### 3.2. Stability and β-Glucuronidase-Dependent Drug Release

**Figure 2:**
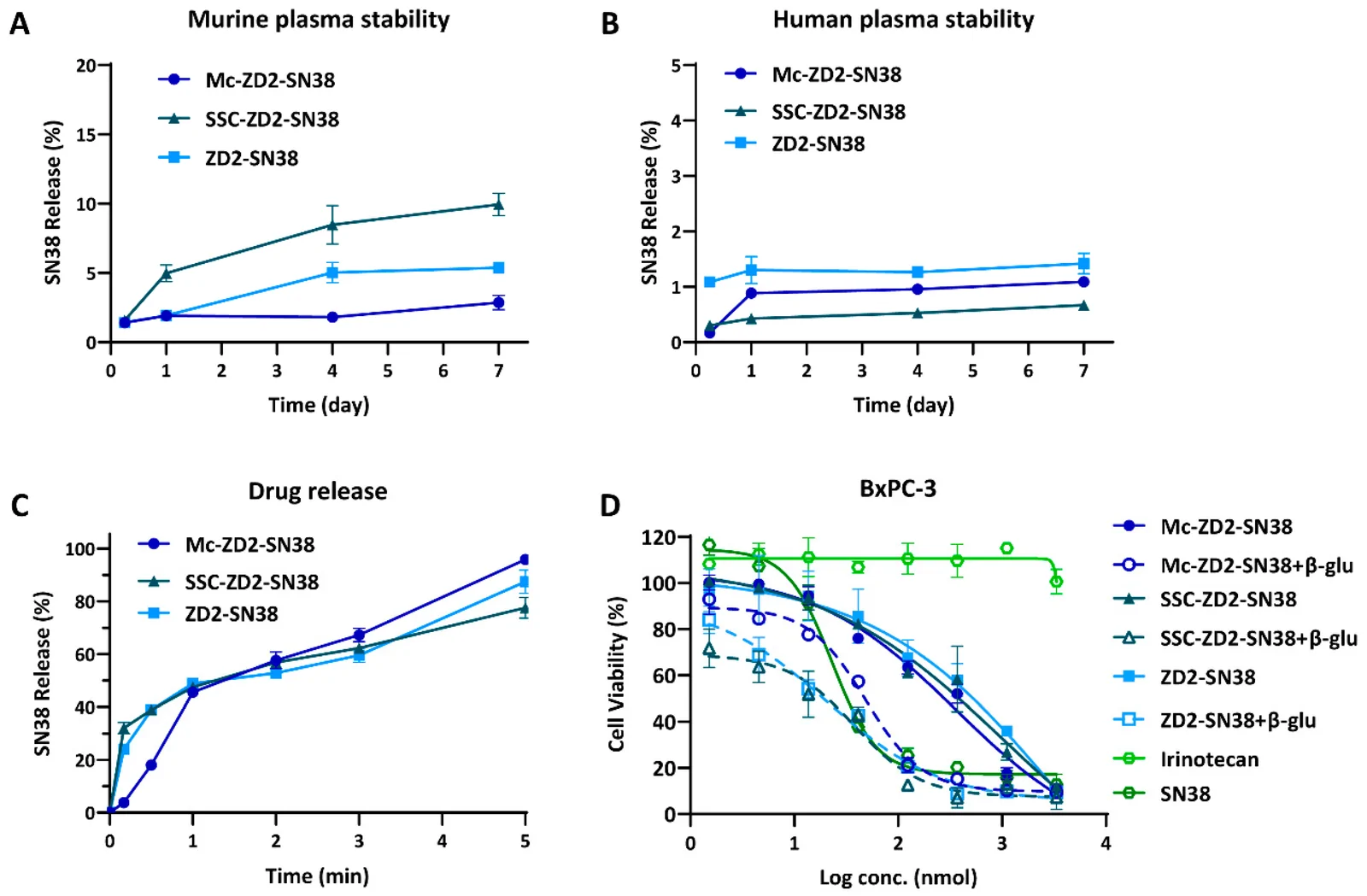
(**A**) Stability of **Mc-ZD2-SN38**, **SSC-ZD2-SN38** and **ZD2-SN38** in murine plasma over 7 days at 37 °C; (**B**) stability of **Mc-ZD2-SN38**, **SSC-ZD2-SN38** and **ZD2-SN38** in human plasma over 7 days at 37 °C; (**C**) kinetics of **SN38** release from **Mc-ZD2-SN38**, **SSC-ZD2-SN38** and **ZD2-SN38** in the presence of β-glucuronidase at 37 °C; (**D**) antiproliferative activities of **Mc-ZD2-SN38**, **SSC-ZD2-SN38** and **ZD2-SN38** with or without β-glucuronidase, as well as irinotecan and **SN38** after 3 days’ treatment in BxPC-3 cells.

In vitro stability assays were conducted in murine and human plasma over 7 days. Following incubation at 37 °C in murine plasma (Figure 2A and Table S1), **Mc-ZD2-SN38** exhibited the highest stability, with **SN38** release below 3%. **ZD2-SN38** and **SSC-ZD2-SN38** demonstrated comparatively lower stability, releasing less than 6% and 10% of **SN38**, respectively. Notably, when incubated under identical conditions in human plasma, all three compounds showed **SN38** release rates below 2% (Figure 2B and Table S2), indicating substantially enhanced stability relative to their performance in murine plasma. This interspecies discrepancy may be attributed to compositional differences in plasma components, but the mechanistic basis for the stability difference between murine and human plasma remains unexplained. These findings demonstrate distinct stability profiles for the three PDC drugs across plasma systems, with human plasma conferring superior stability that may contribute to reduced off-target drug release.

In vitro drug release assays were performed to evaluate enzymatic hydrolysis and subsequent **SN38** release under simulated β-glucuronidase conditions. **Mc-ZD2-SN38**, **SSC-ZD2-SN38** and **ZD2-SN38** were incubated with β-glucuronidase, and **SN38** release was quantified by HPLC analysis. As shown in Figure 2C and Table S3, all three PDCs exhibited **SN38** release exceeding 75% within 5 h. Notably, the incorporation of the ZD2 peptide introduced steric hindrance within the molecular structure, resulting in a modestly slower release rate. However, this structural modification did not substantially impair β-glucuronidase-mediated substrate recognition and cleavage.

The above results show that **Mc-ZD2-SN38**, **SSC-ZD2-SN38** and **ZD2-SN38** can not only maintain high stability in plasma, but also achieve rapid and selective release in the presence of β-glucuronidase. This dual functionality of stable circulation and targeted release serves to minimize systemic exposure of free **SN38** to non-target tissues, thereby reducing off-target toxicity. These characteristics provide a critical foundation for enhancing therapeutic safety and expanding the therapeutic window.

### 3.3. In Vitro Antiproliferative Activity

β-Glucuronidase is highly enriched in solid tumor microenvironments, suggesting that the development of β-glucuronidase-sensitive prodrugs represents a promising strategy for targeted drug activation [32]. We hypothesized that, in the absence of β-glucuronidase, **SN38** release is abrogated, resulting in attenuated cytotoxic potency of PDCs against tumor cells; conversely, effective **SN38** release and subsequent cytotoxic effects are achieved exclusively within β-glucuronidase-rich tumor microenvironments. To evaluate this selective activation mechanism, in vitro anti-proliferative assays were conducted using BxPC-3 (human pancreatic cancer cell line). β-Glucuronidase was exogenously supplemented to the culture medium to simulate the β-glucuronidase-rich tumor microenvironment. As shown in Figure 2D and Table S4, the anti-proliferative activities of **Mc-ZD2-SN38**, **SSC-ZD2-SN38**, and **ZD2-SN38** were markedly lower than **SN38**. However, upon addition of β-glucuronidase, **SN38** was effectively released, and the anti-proliferative effects of all three PDCs became comparable to those of free **SN38**. These findings substantiate that **Mc-ZD2-SN38**, **SSC-ZD2-SN38**, and **ZD2-SN38** possess selective activation capabilities. This enzyme-dependent release mechanism enhances on-target cytotoxicity while minimizing systemic toxicity to normal tissues, thereby improving therapeutic safety.

### 3.4. Pharmacokinetic Analysis

**Figure 3:**
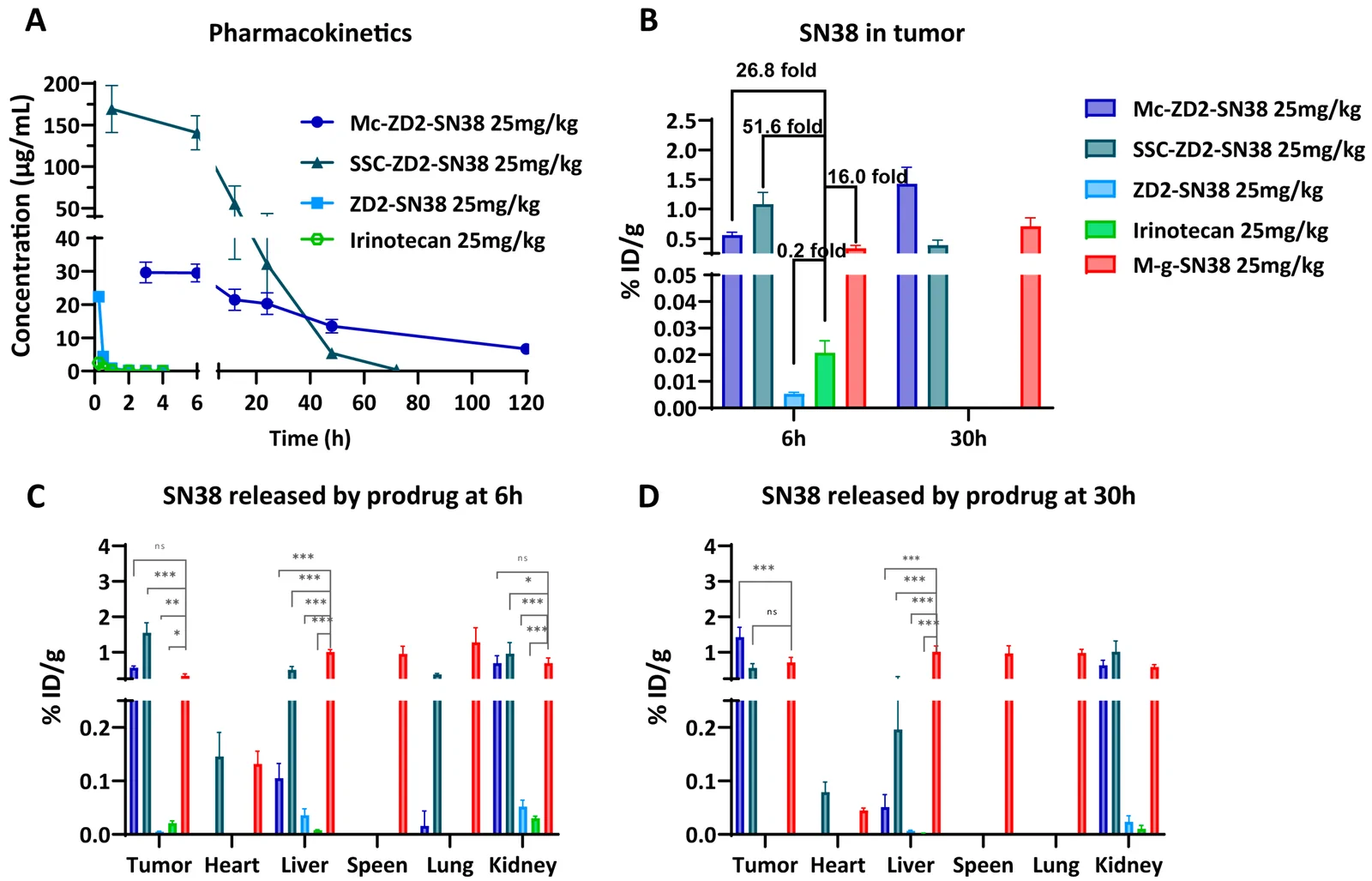
(**A**) The illustration of plasma concentration profiles of **Mc-ZD2-SN38**, **SSC-ZD2-SN38**, **ZD2-SN38** and irinotecan following intravenous administration in mice; (**B**) the concentration of **SN38** within tumor tissue at 6 h and 30 h after the treatment with **Mc-ZD2-SN38**, **SSC-ZD2-SN38**, **ZD2-SN38** and irinotecan; biodistribution of **SN38** in tumor tissues and major organs (%ID/g) at 6 h (**C**) and 30 h (**D**) after administration of **Mc-ZD2-SN38**, **SSC-ZD2-SN38**, **ZD2-SN38** and irinotecan. ns *p* ≥ 0.05, * *p* < 0.05, ** *p* < 0.01, or *** *p* < 0.001 (two-way ANOVA and Dunnett).

The pharmacokinetic profiles of healthy BALB/c mice were evaluated following a single intravenous dose (25 mg/kg) of **Mc-ZD2-SN38**, **SSC-ZD2-SN38**, **ZD2-SN38**, or **irinotecan** via the tail vein (Figure 3A and Table 1). Irinotecan exhibited a half-life of merely 0.52 ± 0.05 h. **ZD2-SN38** displayed a half-life of 0.25 ± 0.01 h, approximately 0.48-fold that of irinotecan. This abbreviated half-life is attributable to the highly hydrophilic ZD2 peptide and β-glucuronide moiety present in its structure, coupled with the absence of albumin-binding functionality. In contrast, **Mc-ZD2-SN38** and **SSC-ZD2-SN38**, both incorporating albumin-binding functionalities, exhibited substantially prolonged half-lives of 52.44 ± 2.41 h and 8.26 ± 0.09 h, respectively, corresponding to 100.8-fold and 15.8-fold increases relative to irinotecan. Notably, the half-life of **Mc-ZD2-SN38** was 6.4-fold greater than that of **SSC-ZD2-SN38**. Compared with **irinotecan**, **Mc-ZD2-SN38** and **SSC-ZD2-SN38** demonstrated significant advantages, with clearance rates reduced by 705.1-fold and 169.9-fold, respectively. Additionally, both albumin-binding PDCs exhibited substantially AUC values, indicating enhanced systemic exposure and favorable conditions for improved therapeutic efficacy.

Collectively, these findings demonstrate that **ZD2-SN38** undergoes rapid elimination in vivo, attributable to the highly hydrophilic structure of both the ZD2 peptide and the β-glucuronide trigger. This rapid clearance represents a common limitation of PDCs, which often necessitates frequent dosing or leads to suboptimal bioavailability, thereby compromising therapeutic efficacy. Conversely, **Mc-ZD2-SN38** and **SSC-ZD2-SN38**, engineered with albumin-binding strategies, acquire macromolecular pharmacokinetic properties. These modifications effectively overcome the limitations of short half-life and rapid clearance while significantly enhancing bioavailability. Notably, covalent albumin-binding PDC **Mc-ZD2-SN38** exhibits superior pharmacokinetic characteristics compared with the noncovalent albumin-binding PDC **SSC-ZD2-SN38**, underscoring its potential advantages in drug delivery system design.

### 3.5. Biodistribution Study

**Mc-ZD2-SN38**, **SSC-ZD2-SN38**, and **ZD2-SN38** all undergo β-glucuronidase-mediated hydrolysis to release **SN38**, thereby eliciting cytotoxic effects against tumor cells. Off-targeted distribution of **SN38** to major organs raises concerns on systemic toxicity and potential tissue damage. Our previous study demonstrated that although **M-g-SN38** achieved superior tumor accumulation and enhanced antitumor efficacy, pronounced off-target accumulation in major organs persisted due to the lack of active targeting moieties [30]. Based on these findings, the ZD2 peptide was employed to confer active targeting capability and further optimize the therapeutic index.

The biodistribution of **Mc-ZD2-SN38**, **SSC-ZD2-SN38**, **ZD2-SN38**, **M-g-SN38** and irinotecan was evaluated at 6 and 30 h post-injection (Figure 3B–D). The percentage of injected dose per gram (%ID/g) of **SN38** in tumors and major organs was determined to assess the potential for enhanced antitumor efficacy and off-target toxicity.

At 6 h post-administration, tumor accumulation of **SN38** in the irinotecan group was 0.021 ± 0.005%ID/g, which declined to undetectable levels by 30 h (Figure 3B). For **ZD2-SN38**, tumor accumulation was markedly lower at 0.005 ± 0.001%ID/g at 6 h (approximately 24% of the irinotecan level), with no detectable **SN38** present at 30 h (Figure 3B). Although the ZD2 peptide confers ECM-targeting capability, the highly hydrophilic structure and rapid metabolism of **ZD2-SN38** result in a short half-life and consequently limited tumor accumulation, thereby restricting its therapeutic potential (Figure 3A and Table 1). In contrast, **M-g-SN38** exhibited substantially higher tumor accumulation at both 6 h (0.336 ± 0.050%ID/g) and 30 h (0.710 ± 0.143%ID/g). However, the prolonged circulation of albumin alone lacks active targeting specificity, leading to widespread systemic distribution of **SN38**, including elevated accumulation in the lungs and spleen, which may contribute to dose-limiting toxicity (Figure 3C,D).

Following administration, **Mc-ZD2-SN38** achieved tumor accumulation levels of 0.562 ± 0.047%ID/g at 6 h and 1.426 ± 0.282%ID/g at 30 h. In contrast, **SSC-ZD2-SN38** exhibited higher early accumulation at 1.084 ± 0.198%ID/g at 6 h, which subsequently declined to 0.391 ± 0.082%ID/g by 30 h (Figure 3B). The more rapid enzymatic release kinetics of **SSC-ZD2-SN38** resulted in an earlier peak tumor accumulation compared with **Mc-ZD2-SN38**, consistent with our previous findings for **M-g-SN38** and **S-g-SN38** [30]. Owing to ZD2 peptide-mediated targeting of EDB-FN in the ECM, **Mc-ZD2-SN38** and **SSC-ZD2-SN38** exhibited significantly reduced **SN38** accumulation in the liver, lungs and spleen compared with **M-g-SN38**. At 6 h post-administration, hepatic **SN38** accumulation in the **Mc-ZD2-SN38** and **SSC-ZD2-SN38** groups was reduced by 9.6-fold and 2.0-fold, respectively, compared with **M-g-SN38**. Similarly, pulmonary **SN38** accumulation was reduced by 79.9-fold and 3.4-fold, respectively. Notably, no detectable **SN38** accumulation was observed in the spleen for either **Mc-ZD2-SN38** or **SSC-ZD2-SN38**, whereas **M-g-SN38** exhibited substantial splenic accumulation (0.957 ± 0.216%ID/g). At 30 h, hepatic accumulation remained markedly lower for **Mc-ZD2-SN38** and **SSC-ZD2-SN38** with no detectable **SN38** in either the spleen or lungs. In contrast, **M-g-SN38** maintained considerable accumulation in the liver (1.014 ± 0.166%ID/g), spleen (0.970 ± 0.219%ID/g) and lungs (0.986 ± 0.101%ID/g) (Figure 3C,D).

**Mc-ZD2-SN38** and **SSC-ZD2-SN38** successfully integrate the dual advantages of prolonged albumin circulation and ZD2 peptide-mediated active targeting. This dual strategy not only overcomes the limitations of rapid metabolism and insufficient tumor accumulation characteristic of PDCs, but also minimizes the risk of non-specific distribution. **Mc-ZD2-SN38** exhibited superior and sustained **SN38** tumor accumulation at 30 h post-administration, suggesting a potential for prolonged therapeutic efficacy. In contrast, **SSC-ZD2-SN38** achieved more rapid tumor accumulation, thereby facilitating the prompt attainment of therapeutically effective concentrations during the early treatment phase.

### 3.6. In Vivo Antitumor Efficacy in the BxPC-3 Xenograft

**Figure 4:**
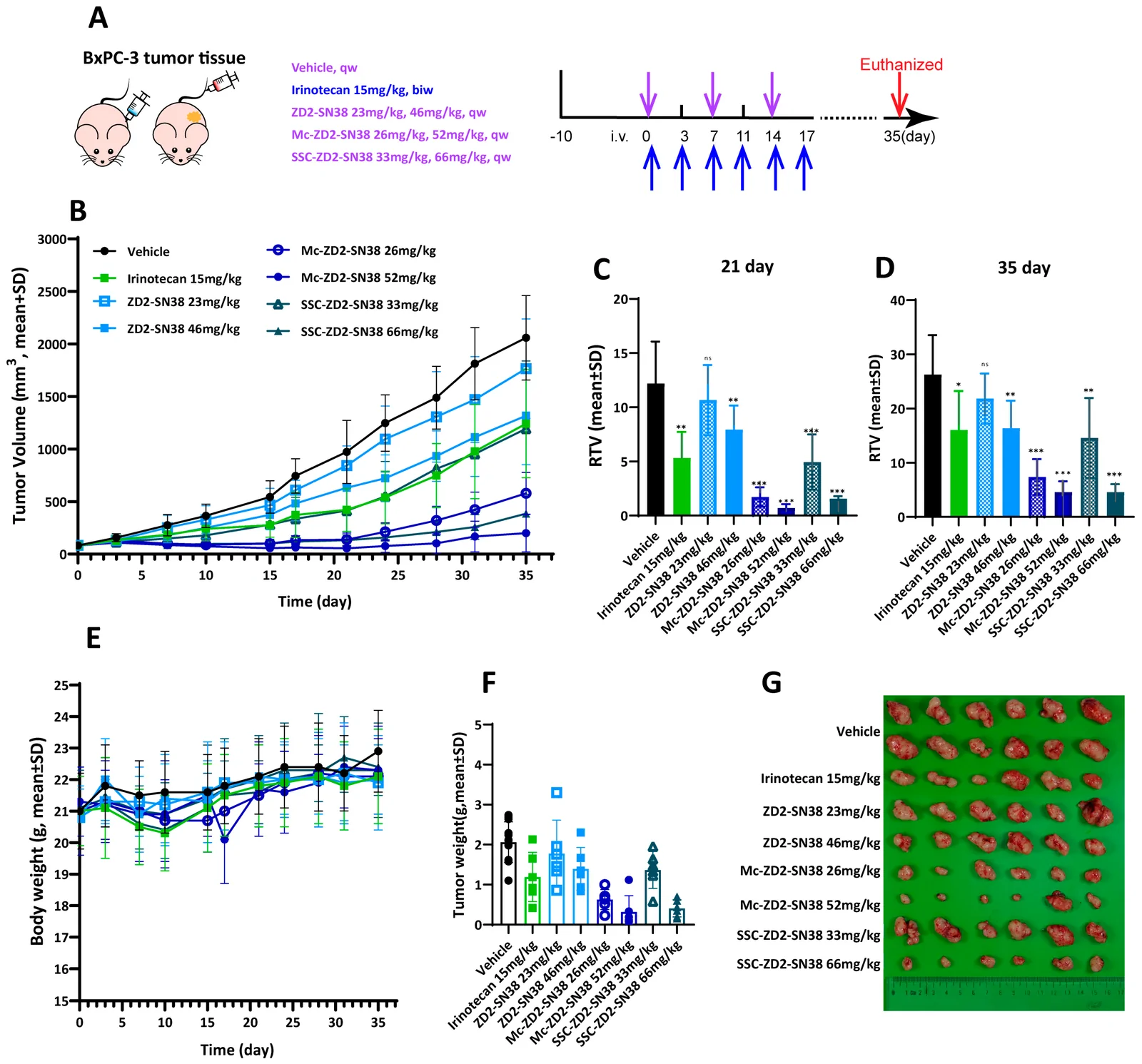
(**A**) Experimental scheme. Treatment regimens: **Mc-ZD2-SN38**, **SSC-ZD2-SN38**, and **ZD2-SN38** were administered once weekly for 3 weeks starting from day 0 (3 doses total); irinotecan was twice weekly for 3 weeks (6 doses total). (**B**) Tumor volume of BxPC-3 xenograft-bearing mice of the 8 groups over 35 days; Relative tumor volume (RTV) of mice bearing BxPC-3 xenografts on day 21 (**C**) and day 35 (**D**), ns *p* ≥ 0.05, * *p* <0.05, ** *p* <0.01, *** *p* <0.001 (Student’s *t*-test vs. Vehicle); (**E**) body weight of the BxPC-3 xenograft-bearing mice of 8 groups over 35 days; (**F**) tumor weight of mice bearing BxPC-3 xenografts on day 35; (**G**) tumor tissues of BxPC-3 xenografts-bearing mice in each group.

The in vivo antitumor efficacy of **Mc-ZD2-SN38**, **SSC-ZD2-SN38**, and **ZD2-SN38** was evaluated in a BxPC-3 xenograft mouse model, with **irinotecan** serving as the positive control. To ensure therapeutic equivalency, the high-dose regimens for all three treatment groups were administered at an equimolar dose corresponding to the established **irinotecan** reference dose of 15 mg/kg, whereas the low-dose regimens corresponded to an equimolar dose of 7.5 mg/kg irinotecan. Specifically, **Mc-ZD2-SN38** was administered at 26 and 52 mg/kg, **SSC-ZD2-SN38** at 33 and 66 mg/kg, and **ZD2-SN38** at 23 and 46 mg/kg. **Mc-ZD2-SN38**, **SSC-ZD2-SN38** and **ZD2-SN38** were administered via intravenous injection once weekly for 3 weeks (total of 3 doses), whereas irinotecan was administered twice weekly for 3 weeks (total of 6 doses). Following the completion of administration, tumor growth was monitored continuously, and all mice were euthanized on day 35 (Figure 4A).

As shown in Figure 4B, the tumor volume of vehicle group increased rapidly over time, whereas all other administration groups exhibited varying degrees of antitumor effects. The irinotecan group partially inhibited tumor growth (Figure 4B), with a T/C value of 43.69% on day 21 (Figure 4C and Table S5). However, tumor regrowth was observed after post-treatment, and the T/C value increased to 61.05% by day 35 (Figure 4D and Table S5). In the low-dose group, **ZD2-SN38** (23 mg/kg) showed no significant inhibitory effect on tumor growth. Although high-dose **ZD2-SN38** (46 mg/kg) partially suppressed tumor growth, the antitumor efficacy was still lower than that of irinotecan, as indicated by T/C values of 65.10% on day 21 and 62.30% on day 35 (Figure 4B–D and Table S5). These findings indicate that, despite the tumor ECM-targeting capability of the ZD2 peptide, the short half-life and limited tumor accumulation of **ZD2-SN38** substantially restrict its therapeutic efficacy. By contrast, **SSC-ZD2-SN38** exhibited enhanced antitumor efficacy. Even at the lower dose (33 mg/kg), which corresponded to half the molar dose and dosing frequency of irinotecan, it achieved a T/C value of 40.54% on day 21 and 56.15% on day 35. This result indicates a stronger inhibitory effect on tumor growth compared with the positive control. The high-dose group (66 mg/kg) showed more pronounced tumor suppression, with the T/C value decreasing to 12.89% on day 21. Although tumor regrowth occurred following treatment discontinuation, the T/C value remained favorable at 17.39% on day 35, demonstrating favorable treatment durability (Figure 4B–D and Table S5). Among all treatment groups, **Mc-ZD2-SN38** exhibited the most prominent antitumor effect. The low-dose (26 mg/kg) and high-dose (52 mg/kg) groups achieved T/C values of 14.10% and 5.71% on day 21, respectively. Following the 2-week observation period, T/C values remained low at 28.00% and 9.23% on day 35, respectively, indicating potent tumor suppression and favorable efficacy durability (Figure 4B–D and Table S5).

It is worth noting that while the developed PDCs structurally liberate **SN38** as the active cytotoxic payload, irinotecan—a clinically authorized water-soluble prodrug of **SN38**—was intentionally selected as the in vivo reference control in Figure 3 and Figure 4.

These results demonstrate that albumin-binding PDCs, particularly **Mc-ZD2-SN38**, exhibit antitumor efficacy comparable to or superior to that of the positive control (Figure 4F,G). Notably, this enhanced therapeutic outcome is achieved with markedly reduced dosing frequency and molar dosage. These advantages are attributable to the favorable pharmacokinetic profile and efficient tumor-targeted accumulation characteristic. Additionally, no treatment-related mortality occurred, and the mice exhibited no significant body weight loss during the in vivo antitumor experiment (Figure 4E).

The superior anti-tumor efficacy of the PDCs was achieved with reduced dosing frequency and a lower cumulative exposure, representing a substantial improvement in dosing convenience and translational safety.

### 3.7. Histological Analysis

**Figure 5:**
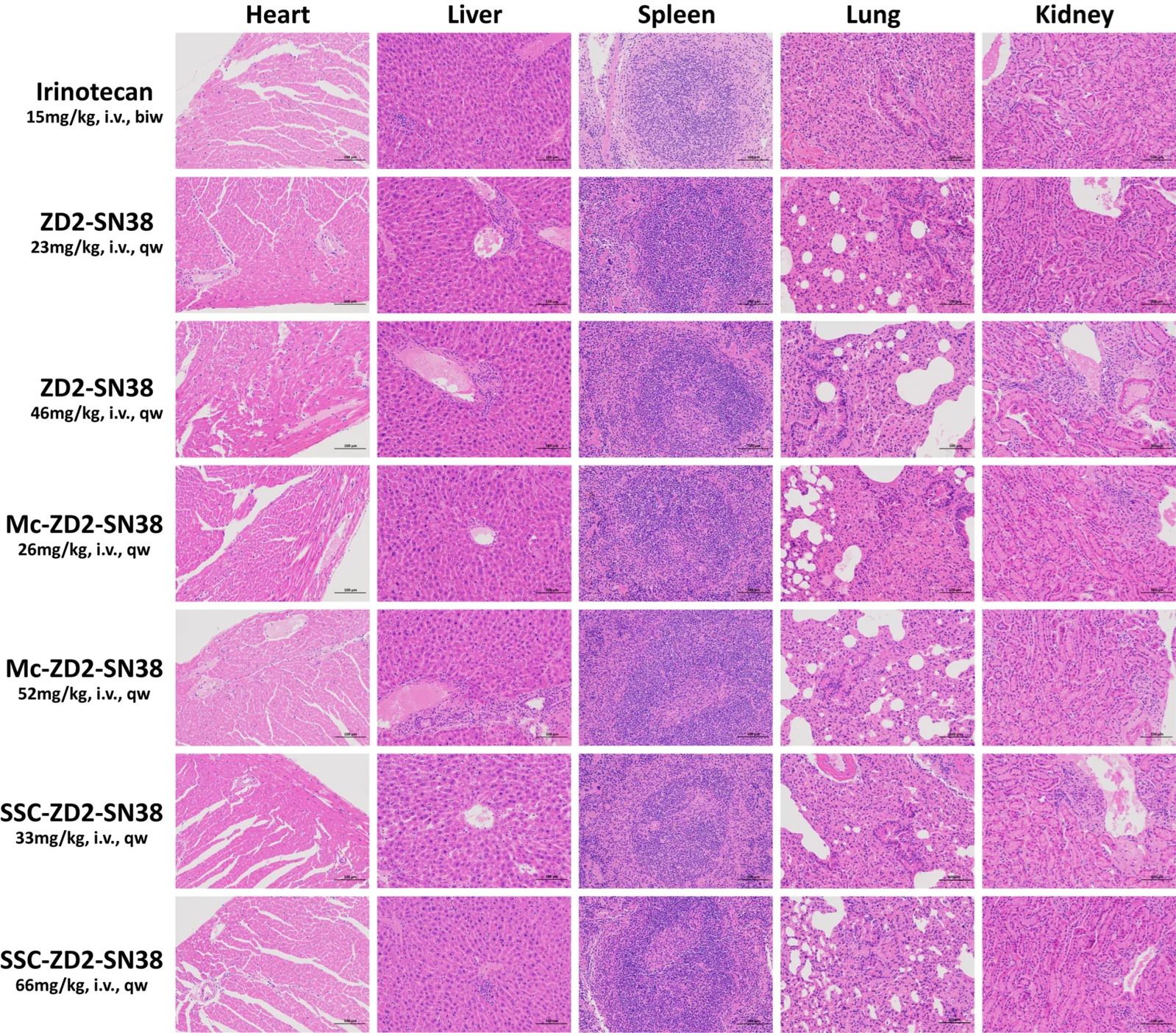
Ex vivo histopathological analysis of major organs through H&E staining (scale bar 100 μm).

Histopathological analysis of major organs through H&E staining was subsequently performed to reveal differences in tissue damage (Figure 5). Alveolar collapse resulting from CO_2_ euthanasia was observed uniformly across all groups, yet this artifact does not compromise the evaluation of drug-induced pathological changes. In the irinotecan group, inflammatory cell infiltration was observed in the lungs, suggestive of drug-associated pulmonary injury. In contrast, mice from the PDC treatment groups exhibited no significant inflammatory cell infiltration, fibrosis, or other overt pathological lesions in the major organs. Collectively, these results indicate that albumin-binding PDCs did not induce any discernible drug-related pathological damage in major organs throughout the treatment period, underscoring its favorable preliminary safety and biocompatibility at the therapeutic dose.

## 4. Conclusions

In conclusion, we have successfully developed two albumin-binding PDCs, designated **Mc-ZD2-SN38** and **SSC-ZD2-SN38**, alongside the non-albumin-binding PDC ZD2-SN38. All three PDCs target EDB-FN in the tumor ECM via the ZD2 peptide. The albumin-binding PDCs, **Mc-ZD2-SN38** and **SSC-ZD2-SN38**, were specifically designed to confer prolonged circulation upon the drug delivery system, thereby addressing the inadequate tumor accumulation that limits conventional PDCs.

In vitro stability assays demonstrated that all three PDCs remained stable in both murine and human plasma, with **SN38** release specifically triggered by β-glucuronidase. Furthermore, in vitro antiproliferation assays confirmed that significant tumor cell growth inhibitory activity was observed only upon β-glucuronidase-mediated **SN38** release.

Pharmacokinetic studies revealed that **ZD2-SN38** exhibited a markedly short elimination half-life, even shorter than that of irinotecan. In contrast, the half-lives of **Mc-ZD2-SN38** and **SSC-ZD2-SN38** were substantially prolonged, representing 100.8-fold and 15.8-fold increases relative to **irinotecan**, respectively. Notably, the covalent albumin-binding strategy (**Mc-ZD2-SN38**) proved superior to the non-covalent approach (**SSC-ZD2-SN38**) in enhancing pharmacokinetic properties.

Biodistribution studies revealed low tumor **SN38** accumulation for both **ZD2-SN38** and irinotecan, whereas **Mc-ZD2-SN38** and **SSC-ZD2-SN38** achieved markedly elevated intratumoral **SN38** levels. The ZD2 peptide-mediated targeting of EDB-FN enabled minimal off-target accumulation for both albumin-binding PDCs, indicative of an improved safety profile and an expanded therapeutic window.

BxPC-3 cell line and tumor xenograft have been documented to express EDB-FN, making it a highly reliable and well-established preclinical model for investigating ZD2 peptide-mediated active molecular targeting [33,34]. Thus, the tumor accumulation and selective uptake observed in our study firmly validate the target-specific binding capability of **Mc-ZD2-SN38** and **SSC-ZD2-SN38** within our experimental system.

In the BxPC-3 xenograft model, **Mc-ZD2-SN38** and **SSC-ZD2-SN38** achieved profound and sustained tumor suppression relative to irinotecan with less frequent administration and lower cumulative exposure. Histopathological analysis further confirmed the absence of significant drug-related pathological lesions following treatment with either **Mc-ZD2-SN38** or **SSC-ZD2-SN38**, indicating a highly favorable systemic biocompatibility profile at the therapeutic dose. However, it should be noted that because the non-targeted M-g-SN38 conjugate was excluded from this histological cohort, direct tissue-level comparative toxicity dynamics between the targeted and non-targeted constructs remain to be established in future investigations.

Several limitations should be noted in the current study. First, the present study employed the same albumin-binding moieties, namely the maleimide and semaglutide side chain, which were previously validated in our studies of **M-g-SN38** and **S-g-SN38**. Consistent with our previous findings, these newly synthesized ZD2-containing peptide–drug conjugates exhibited markedly prolonged in vivo circulation, suggesting effective albumin binding. However, despite these pharmacokinetic data, direct in vitro biophysical characterization of their interaction with plasma albumin was not performed in the present study.

Second, pharmacokinetic evaluation was conducted only at 25 mg/kg, whereas efficacy studies were performed using molar-equivalent doses ranging from 26 to 66 mg/kg. Because dose proportionality and pharmacokinetic linearity across this dose range have not been characterized, the pharmacokinetic findings should not be directly extrapolated to the higher efficacy dose levels.

Third, our biodistribution study demonstrated **Mc-ZD2-SN38** and **SSC-ZD2-SN38** displayed biodistribution patterns markedly different from the non-targeted control **M-g-SN38**, with significantly greater BxPC-3 tumor accumulation observed for both ZD2-containing conjugates. These findings are consistent with a substantial contribution of ZD2-mediated active tumor targeting. However, in the absence of competitive blocking experiments, the contribution of passive tumor accumulation through the EPR effect cannot be definitively excluded.

Although the standard subcutaneous BxPC-3 model successfully validated the EDB-FN targeting capability and initial therapeutic efficacy of our PDCs, it represents a simplified platform with inherent limitations that could affect the clinical interpretation of the results. Specifically, it lacks the dense desmoplastic stroma, high interstitial fluid pressure, and poor vascularization that characterize human pancreatic ductal adenocarcinoma (PDAC). In clinical scenarios, these microenvironmental barriers can severely impede drug delivery and deep tumor penetration. Therefore, the robust tumor accumulation and therapeutic outcomes observed in this subcutaneous model might be optimized or partially overestimated compared to actual clinical outcomes. To better translate these findings, future studies employing orthotopic tumor models or patient-derived xenograft (PDX) models are warranted to thoroughly evaluate the therapeutic potential of **Mc-ZD2-SN38** and **SSC-ZD2-SN38** under more clinically relevant pathological conditions.

Collectively, these findings underscore that the pharmacokinetic limitations of conventional PDCs were successfully overcome through the incorporation of albumin-binding strategies. The albumin-binding PDC delivery platform provides a compelling rationale and experimental foundation for the development of the next generation of highly efficacious, low-toxicity targeted antitumor agents.

## Figures and Tables

**Table 1:** Pharmacokinetic parameters of **Mc-ZD2-SN38**, **SSC-ZD2-SN38**, **ZD2-SN38** and irinotecan.

	Mc-ZD2-SN38	SSC-ZD2-SN38	ZD2-SN38	Irinotecan
Cmax (μg/mL)	29.65 ± 3.11	169.21 ± 28.3	22.41 ± 0.24	2.45 ± 0.16
t_1/2_ (h)	52.44 ± 2.41	8.26 ± 0.09	0.25 ± 0.01	0.52 ± 0.05
CL (mL/h)	6.79 ± 1.45	28.18 ± 5.74	6982.93 ± 88.15	4788.60 ± 129.32
AUC_(0–t)_ (h·mg/mL)	1623.65 ± 98.47	2405.84 ± 67.15	5.41 ± 0.46	1.16 ± 0.08

## Data Availability

Data are available upon request to the corresponding authors.

## Supplementary Materials

The following supporting information can be downloaded at https://www.mdpi.com/article/10.3390/pharmaceutics18081028/s1; HPLC method, preparations and prescriptions are provided in the Supplementary Materials. Table S1: Stability of **Mc-ZD2-SN38**, **SSC-ZD2-SN38** and **ZD2-SN38** in murine plasma over a span of 7 days at 37 °C. Table S2: Stability of **Mc-ZD2-SN38**, **SSC-ZD2-SN38** and **ZD2-SN38** in human plasma over a span of 7 days at 37 °C. Table S3: Kinetics of **SN38** release from **Mc-ZD2-SN38**, **SSC-ZD2-SN38** and **ZD2-SN38** in the presence of β-glucuronidase at 37 °C. Table S4: IC50 values for **Mc-ZD2-SN38**, **SSC-ZD2-SN38** and **ZD2-SN38**, both with or without β-glucuronidase, as well as irinotecan and **SN38** after 3 days’ treatment in BxPC-3 cells. Table S5: T/C (%) of mice bearing BxPC-3 xenografts. Figure S1: ^1^H NMR spectra of **SC2**. Figure S2: ^1^H NMR spectra of **3**. Figure S3: ^1^H NMR spectra of **4**. Figure S4: ^1^H NMR spectra of **5**. Figure S5: ^1^H NMR spectra of **Mc-ZD2-SN38**. Figure S6: ^1^H NMR spectra of **SSC-ZD2-SN38**. Figure S7: ^1^H NMR spectra of **ZD2-SN38**. Figure S8: HRMS spectra of **Mc-ZD2-SN38**. Figure S9: HRMS spectra of **SSC-ZD2-SN38**. Figure S10: HRMS spectra of **ZD2-SN38**. Figure S11: HPLC spectra of **Mc-ZD2-SN38**. Figure S12: HPLC spectra of **SSC-ZD2-SN38**. Figure S13: HPLC spectra of **ZD2-SN38**.

## Abbreviation

PDAC	pancreatic ductal adenocarcinoma
ECM	extracellular matrix
EDB-FN	extra domain B fibronectin
SN38	7-ethyl-10-hydroxycamptothecin
PDC	peptide drug conjugate
TME	tumor microenvironment
ZD2 peptide	sequence: TVRTSAD, CTVRTSADC
cCPP	a cyclic cell penetrating peptide
CBT	cabazitaxel
EDBp peptide	sequence: AVRTSAD
β-glu	β-glucuronidase
RTV	relative tumor volume
T/C	relative tumor proliferation rate
PBS	phosphate-buffered saline
HPLC	high performance liquid chromatography
H&E	Hematoxylin and Eosin
