# Supplementary material for: Design, Synthesis and Biological Evaluation of Novel SN38 Based Albumin-Binding Peptide–Drug Conjugates in Pancreatic Ductal Adenocarcinoma
Source: Pharmaceutics. 2026 Aug 19;18(8):1028. doi: 10.3390/pharmaceutics18081028 (PMC13517058; doi:10.3390/pharmaceutics18081028)
Supplement: Supplementary file 1 [file pharmaceutics-18-01028-s001.zip › pharmaceutics-4374012-supplementary.pdf]

**Design, Synthesis and Biological Evaluation of Novel SN38 Based Albumin-Binding Peptide–Drug Conjugates in Pancreatic Ductal Adenocarcinoma**

Yi Su [1,2,†], Yingxin Lu [3,†], Jiahui Yu [3], Chen Jin [4], Yi Chen [2,*], Wei Lu [3,*] and Jian Ding [1,2]

[1] School of Chinese Materia Medica, Nanjing University of Chinese Medicine, Nanjing 210023, China; ysu@simm.ac.cn (Y.S.); jding@simm.ac.cn (J.D.)

[2] State Key Laboratory of Drug Research, Shanghai Institute of Materia Medica, Chinese Academy of Sciences, Shanghai 201203, China

[3] School of Chemistry and Molecular Engineering, East China Normal University, 3663 North Zhongshan Road, Shanghai 200062, China; luyingxin_roy@163.com (Y.L.); jhyu@sist.ecnu.edu.cn (J.Y.)

[4] Shanghai Tekanbio Pharm-Tech Co., Ltd., Room 4001, Floor 4, Unit 3, Building 8, No. 160, Basheng Road, China (Shanghai) Pilot Free Trade Zone, Shanghai 200131, China; jinc@tekanbio.com

***Correspondence**: ychen@simm.ac.cn (Y.C.); wlu@chem.ecnu.edu.cn (W.L.)

[†]These authors contributed equally to this work.

## Content

**Content** .......... 1
**1. HPLC Method** .......... 2
**2. Preparations and prescriptions** .......... 2
**3. Tables** .......... 4
  **Table S1** .......... 4
  **Table S2** .......... 4
  **Table S3** .......... 5
  **Table S4** .......... 5
  **Table S5** .......... 6
**4. $^1$H NMR, HRMS and HPLC** .......... 7
  **Figure S1** .......... 7
  **Figure S2** .......... 7
  **Figure S3** .......... 8
  **Figure S4** .......... 8
  **Figure S5** .......... 9
  **Figure S6** .......... 9
  **Figure S7** .......... 8

**Figure S8** ........................................................................................... 9
**Figure S9** ........................................................................................... 9
**Figure S10** ......................................................................................... 9
**Figure S11** ......................................................................................... 10
**Figure S12** ......................................................................................... 10
**Figure S13** ......................................................................................... 11

## 1. HPLC Method

Agilent ZORBAX SB-C18 column (250 × 4.6 mm, 5 μm) was performed in a gradient system, with detection wavelengths set at 254 nm and 360 nm and a column temperature maintained at 40 °C. Mobile phase A consisted of 0.1% (v/v) trifluoroacetic acid in water. Mobile phase B was acetonitrile. The gradient elution program is detailed in the table below:

| Time (min) | Mobile phase B (%) |
|---|---|
| 0.1 | 5 |
| 15.0 | 95 |
| 20.0 | 95 |
| 20.1 | 5 |
| 30.0 | 5 |

## 2. Preparations and prescriptions

| | |
|---|---|
| **Mc-ZD2-SN38** | 1% DMSO+ 99% saline |
| **SSC-ZD2-SN38** | 1% DMSO+ 99% saline (10% HP-β-CD in saline) |
| **ZD2-SN38** | 1% DMSO+ 99% saline |
| **Irinotecan** | 3% DMSO+ 97% saline |

## 3. Tables

**Table S1**

Stability of **Mc-ZD2-SN38**, **SSC-ZD2-SN38** and **ZD2-SN38** in murine plasma over a span of 7 days at 37 °C.

| Time (day) | **SN38** release % in murine plasma | | |
|---|---|---|---|
| | **Mc-ZD2-SN38** | **SSC-ZD2-SN38** | **ZD2-SN38** |
| 0.25 | 1.41 ± 0.02 | 1.58 ± 0.05 | 1.43 ± 0.03 |
| 1 | 1.91 ± 0.21 | 4.97 ± 0.60 | 1.93 ± 0.33 |
| 4 | 1.81 ± 0.18 | 8.48 ± 1.39 | 5.02 ± 0.73 |
| 7 | 2.85 ± 0.53 | 9.93 ± 0.82 | 5.38 ± 0.24 |

**Table S2**

Stability of **Mc-ZD2-SN38**, **SSC-ZD2-SN38** and **ZD2-SN38** in human plasma over a span of 7 days at 37 °C.

| Time (day) | **SN38** release % in human plasma | | |
|---|---|---|---|
| | **Mc-ZD2-SN38** | **SSC-ZD2-SN38** | **ZD2-SN38** |
| 0.25 | 0.17 ± 0.06 | 0.30 ± 0.07 | 1.08 ± 0.02 |
| 1 | 0.88 ± 0.02 | 0.43 ± 0.06 | 1.30 ± 0.24 |
| 4 | 0.96 ± 0.06 | 0.53 ± 0.06 | 1.26 ± 0.05 |
| 7 | 1.09 ± 0.06 | 0.67 ± 0.03 | 1.42 ± 0.19 |

**Table S3**

Kinetics of **SN38** release from **Mc-ZD2-SN38**, **SSC-ZD2-SN38** and **ZD2-SN38** in the presence of β-glucuronidase at 37 °C.

| Time (min) | **SN38** release % incubated with β-glu | | |
|---|---|---|---|
| | **Mc-ZD2-SN38** | **SSC-ZD2-SN38** | **ZD2-SN38** |
| 0.2 | 3.77 ± 0.06 | 32.05 ± 2.12 | 24.05 ± 1.99 |
| 0.5 | 18.06 ± 1.74 | 38.92 ± 1.14 | 38.91 ± 0.80 |
| 1.0 | 45.52 ± 0.59 | 47.47 ± 1.30 | 48.91 ± 0.21 |
| 2.0 | 57.64 ± 3.14 | 56.87 ± 0.81 | 52.83 ± 1.39 |
| 3.0 | 67.27 ± 2.59 | 62.27 ± 0.90 | 59.57 ± 2.74 |
| 5.0 | 95.88 ± 1.20 | 77.60 ± 3.96 | 87.46 ± 4.41 |

**Table S4**

IC50 values for **Mc-ZD2-SN38**, **SSC-ZD2-SN38** and **ZD2-SN38**, both with or without β-glucuronidase, as well as **irinotecan** and **SN38** after 3 days' treatment in BxPC-3 cells.

| IC50 (nM) | | IC50 (nM) | |
|---|---|---|---|
| **Mc-ZD2-SN38** | 270.2 | **Mc-ZD2-SN38**+β-Glu | 46.69 |
| **SSC-ZD2-SN38** | 349.5 | **SSC-ZD2-SN38**+β-Glu | 14.54 |
| **ZD2-SN38** | 436.3 | **ZD2-SN38**+β-Glu | 19.35 |
| **Irinotecan** | >3333 | **SN38** | 51.59 |

## Table S5

T/C (%) of mice bearing BxPC-3 xenografts.

| Group | T/C（%） | | | | | | | | | | |
|---|---|---|---|---|---|---|---|---|---|---|---|
| | d0 | d3 | d7 | d10 | d15 | d17 | d21 | d24 | d28 | d31 | d35 |
| **Irinotecan 15mg/kg** | 100.00 | 84.07 | 66.92 | 65.80 | 50.72 | 49.10 | 43.69 | 43.53 | 52.01 | 55.99 | 61.05 |
| **ZD2-SN38 23mg/kg** | 100.00 | 84.35 | 86.84 | 87.71 | 85.51 | 81.28 | 87.45 | 84.73 | 83.58 | 78.28 | 83.11 |
| **ZD2-SN38 46mg/kg** | 100.00 | 78.33 | 62.97 | 68.04 | 66.27 | 62.22 | 65.10 | 56.92 | 62.52 | 60.69 | 62.30 |
| **Mc-ZD2-SN38 26mg/kg** | 100.00 | 78.89 | 35.68 | 25.40 | 18.09 | 17.91 | 14.10 | 17.29 | 20.84 | 22.86 | 28.00 |
| **Mc-ZD2-SN38 52mg/kg** | 100.00 | 71.73 | 31.30 | 20.80 | 10.53 | 8.32 | 5.71 | 5.95 | 6.67 | 8.75 | 9.23 |
| **SSC-ZD2-SN38 33mg/kg** | 100.00 | 69.26 | 51.88 | 47.26 | 47.18 | 41.17 | 40.54 | 41.16 | 51.39 | 50.37 | 56.15 |
| **SSC-ZD2-SN38 66mg/kg** | 100.00 | 71.59 | 30.58 | 25.92 | 17.39 | 14.90 | 12.89 | 11.85 | 13.33 | 13.32 | 17.39 |

## 4. $^1$H NMR, HRMS and HPLC

## Figure S1

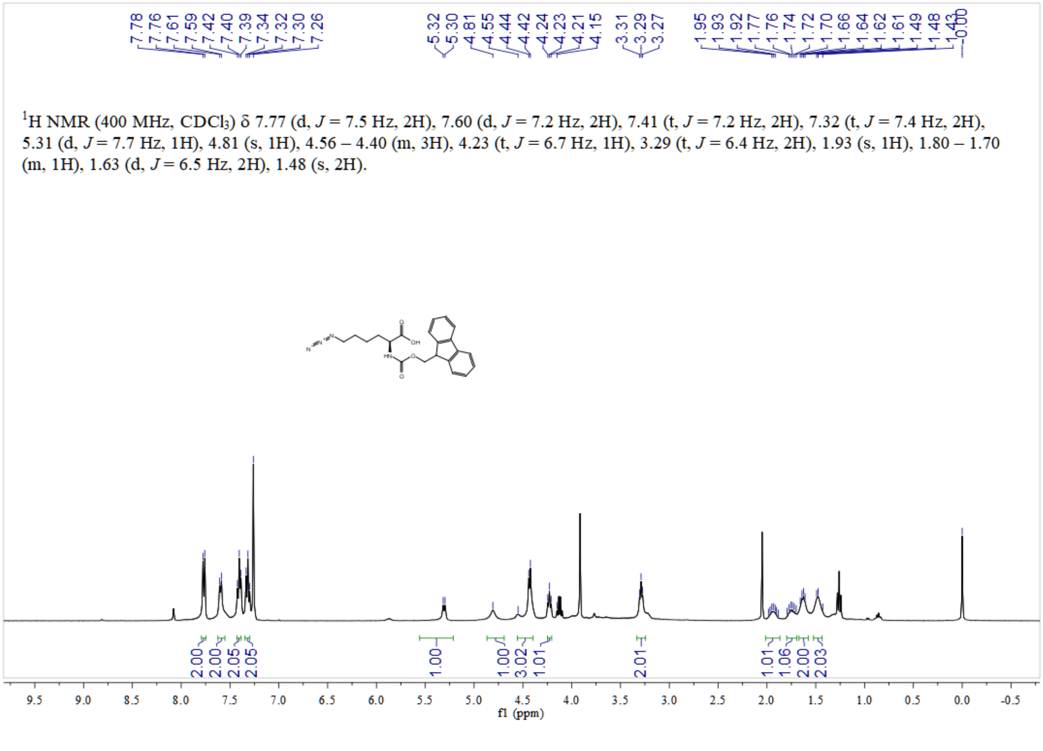


$^1$H NMR spectra of **SC2**

## Figure S2

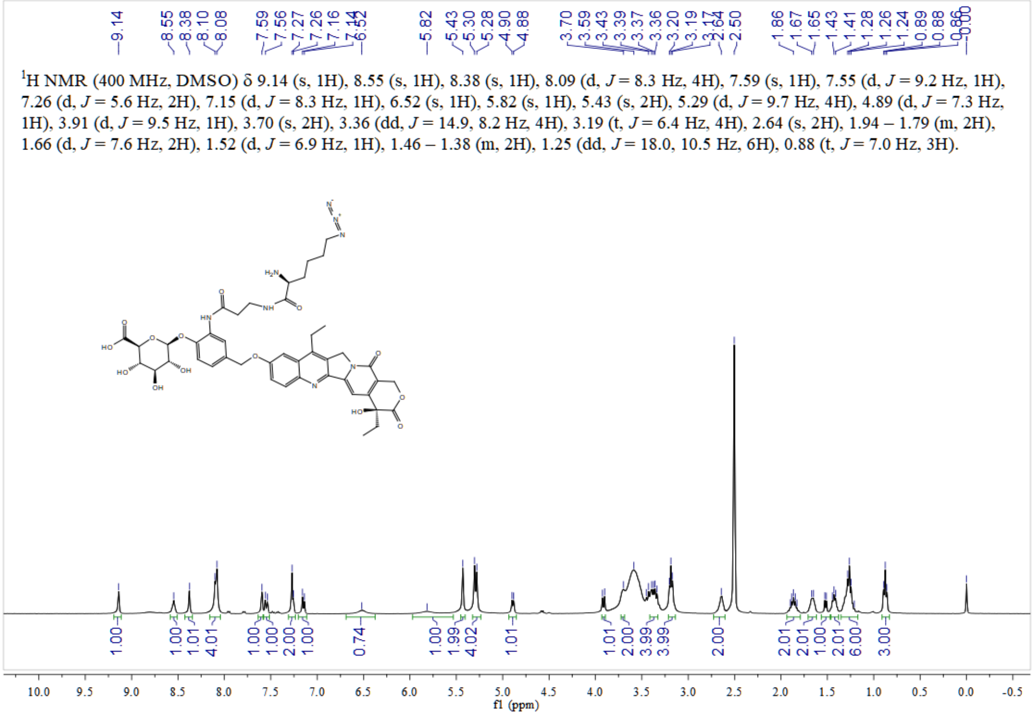


$^1$H NMR spectra of **3**

## Figure S3

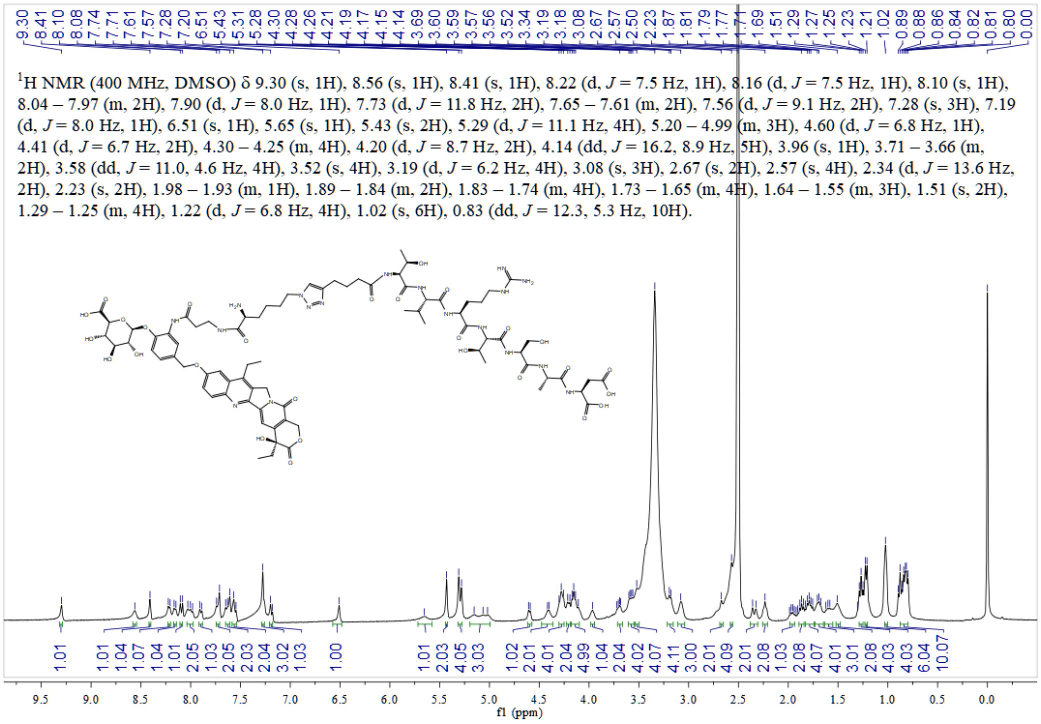


$^1$H NMR spectra of **4**

**Figure S4**

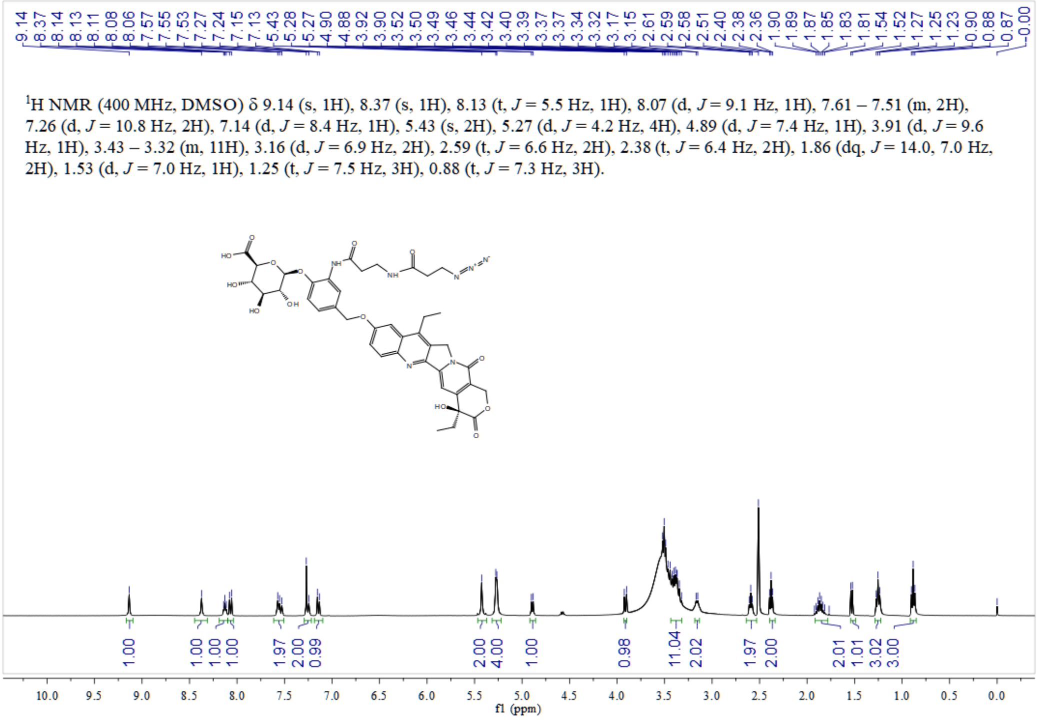


$^{1}$H NMR spectra of **5**

**Figure S5**

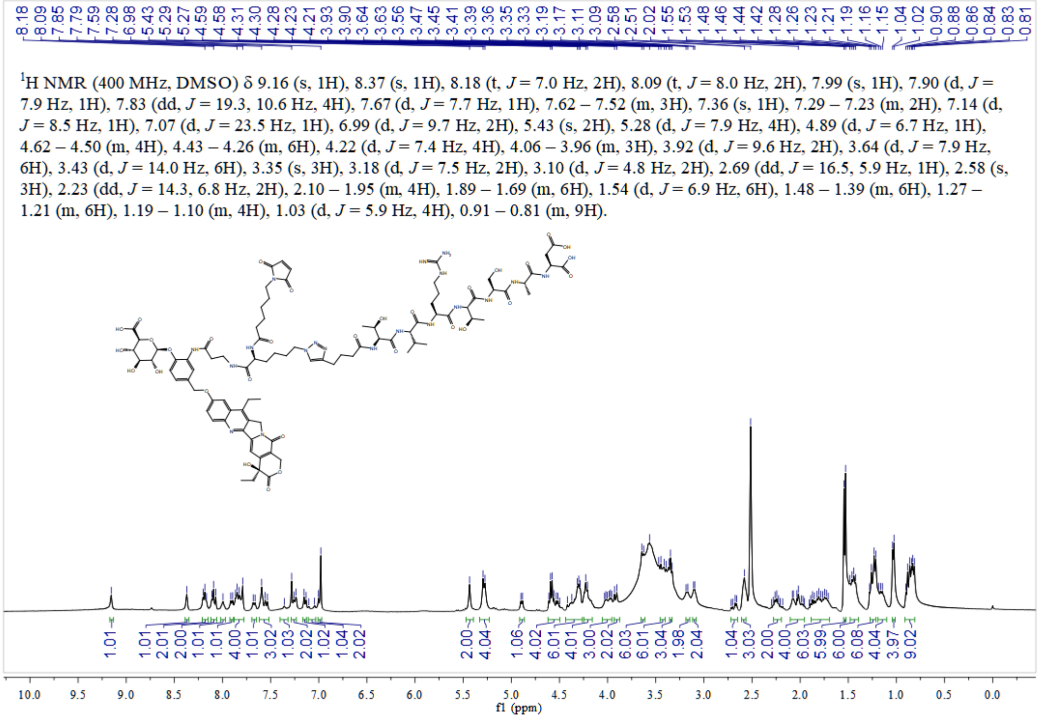


$^{1}$H NMR spectra of **Mc-ZD2-SN38**

## Figure S6

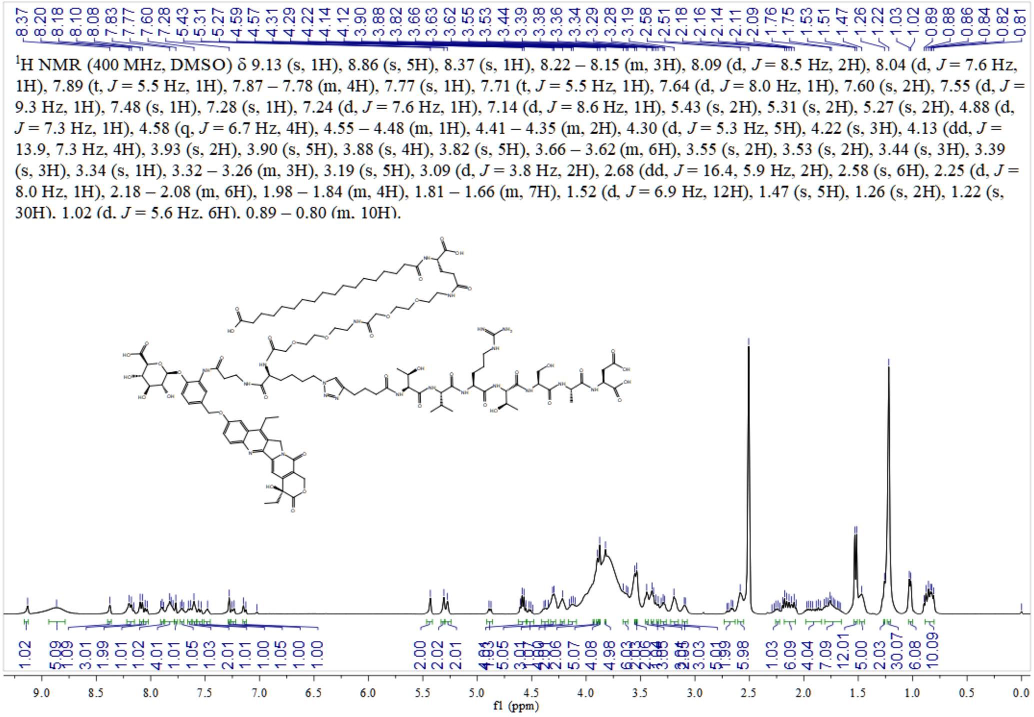


$^{1}$H NMR spectra of **SSC-ZD2-SN38**

## Figure S7

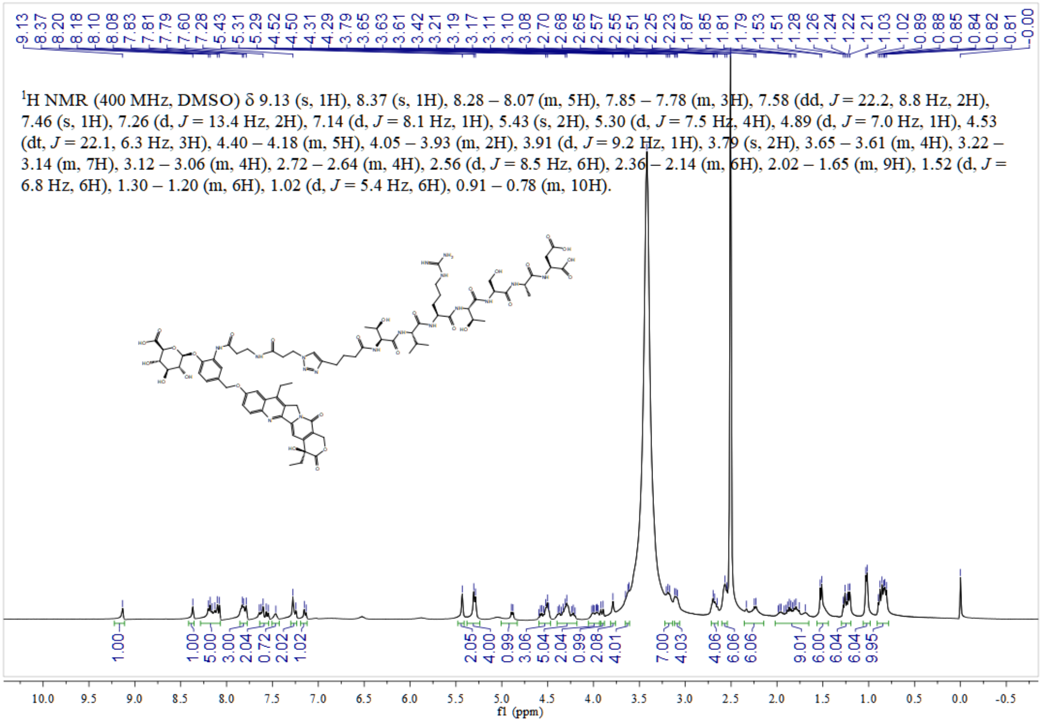


$^{1}$H NMR spectra of **ZD2-SN38**

## Figure S8

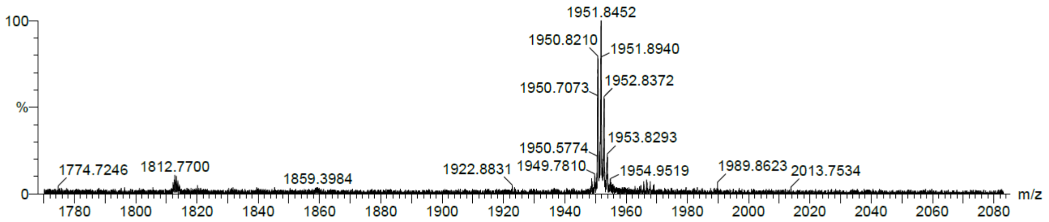


HRMS spectra of **Mc-ZD2-SN38**

## Figure S9

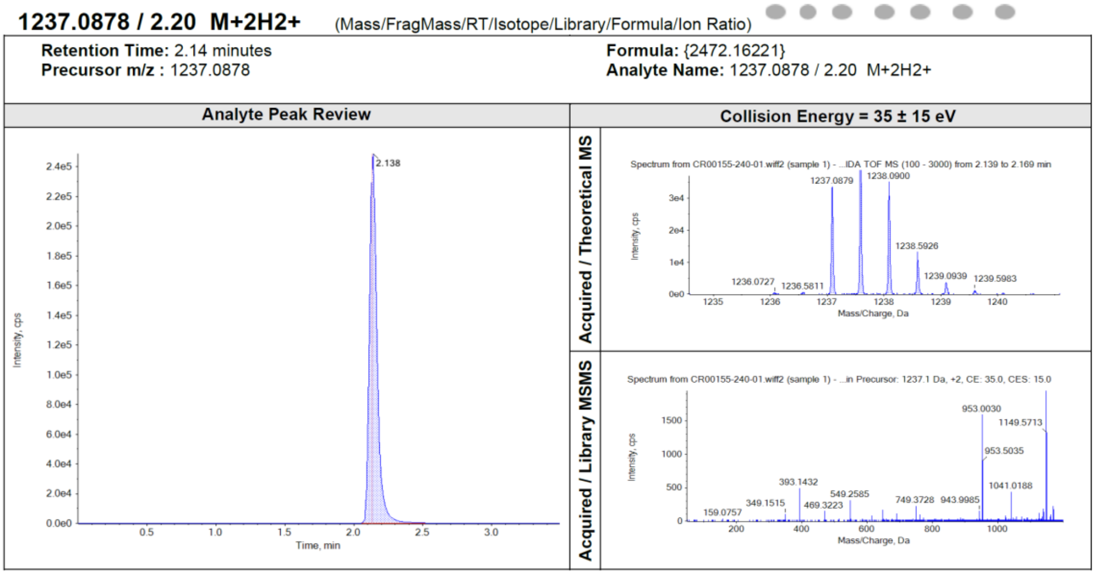


HRMS spectra of **SSC-ZD2-SN38**

## Figure S10

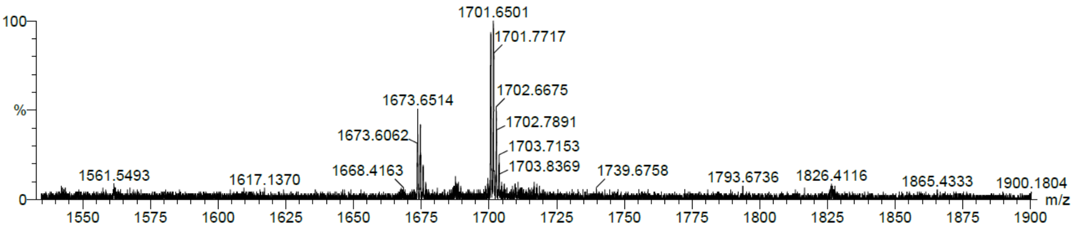


HRMS spectra of **ZD2-SN38**

## Figure S11

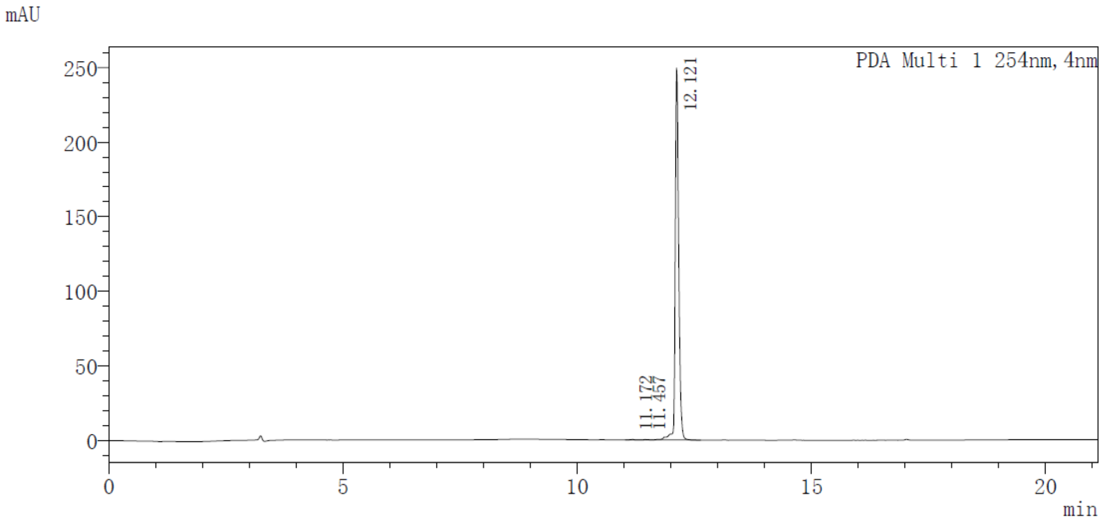


Mc-ZD2-SN38.lcd

PDA Ch1 254nm

| Peak | RetTime | Area | Height | Area% |
|---|---|---|---|---|
| 1 | 11.172 | 1504 | 227 | 0.117 |
| 2 | 11.457 | 2693 | 281 | 0.210 |
| 3 | 12.121 | 1276117 | 249189 | 99.672 |
| Total | | 1280314 | 249696 | 100.000 |

HPLC spectra of **Mc-ZD2-SN38**

## Figure S12

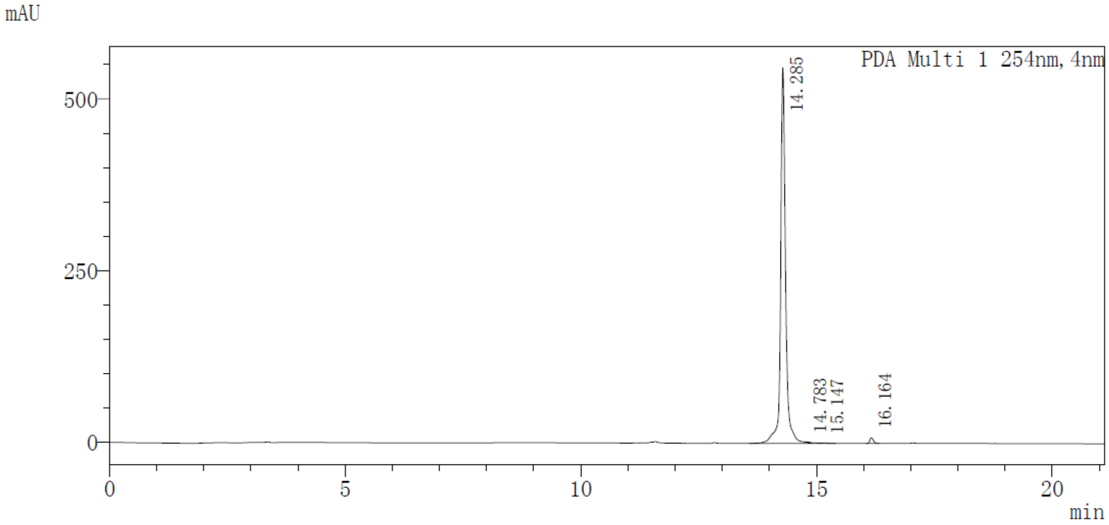


SSC-ZD2-SN38.lcd

PDA Ch1 254nm

| Peak | RetTime | Area | Height | Area% |
|---|---|---|---|---|
| 1 | 14.285 | 3975871 | 546577 | 98.782 |
| 2 | 14.783 | 2343 | 528 | 0.058 |
| 3 | 15.147 | 3052 | 477 | 0.076 |
| 4 | 16.164 | 43638 | 8413 | 1.084 |
| Total | | 4024903 | 555995 | 100.000 |

HPLC spectra of **SSC-ZD2-SN38**

## Figure S13

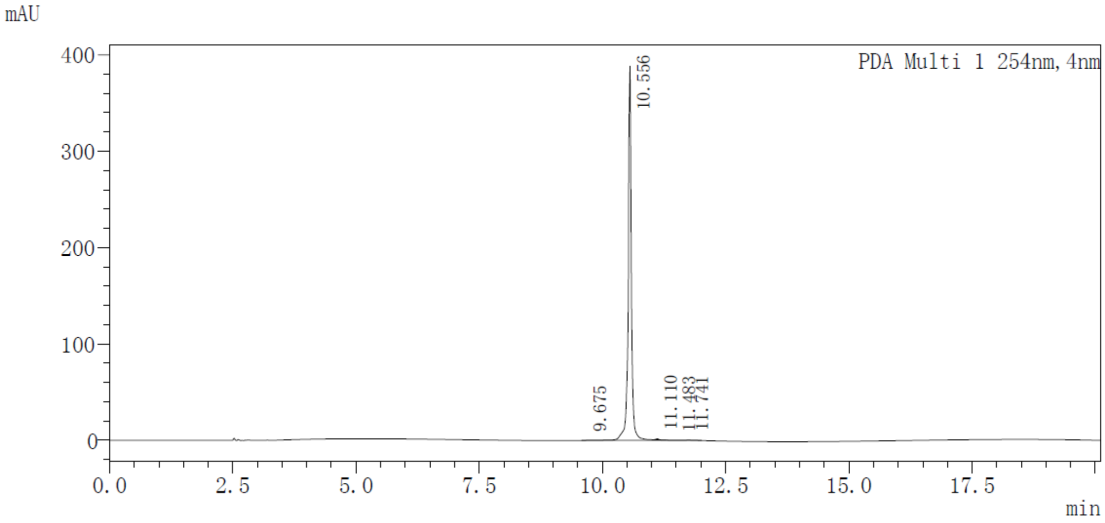


ZD2-SN38.lcd

PDA Ch1 254nm

| Peak | RetTime | Area | Height | Area% |
|---|---|---|---|---|
| 1 | 9.675 | 111 | 12 | 0.006 |
| 2 | 10.556 | 1738491 | 388238 | 99.607 |
| 3 | 11.110 | 4993 | 1141 | 0.286 |
| 4 | 11.483 | 191 | 32 | 0.011 |
| 5 | 11.741 | 1564 | 393 | 0.090 |
| Total | | 1745349 | 389816 | 100.000 |

HPLC spectra of **ZD2-SN38**
